# Insights into the genetic epidemiology of Crohn's and rare diseases in the Ashkenazi Jewish population

**DOI:** 10.1371/journal.pgen.1007329

**Published:** 2018-05-24

**Authors:** Manuel A. Rivas, Brandon E. Avila, Jukka Koskela, Hailiang Huang, Christine Stevens, Matti Pirinen, Talin Haritunians, Benjamin M. Neale, Mitja Kurki, Andrea Ganna, Daniel Graham, Benjamin Glaser, Inga Peter, Gil Atzmon, Nir Barzilai, Adam P. Levine, Elena Schiff, Nikolas Pontikos, Ben Weisburd, Monkol Lek, Konrad J. Karczewski, Jonathan Bloom, Eric V. Minikel, Britt-Sabina Petersen, Laurent Beaugerie, Philippe Seksik, Jacques Cosnes, Stefan Schreiber, Bernd Bokemeyer, Johannes Bethge, International IBD Genetics Consortium, NIDDK IBD Genetics Consortium, T2D-GENES Consortium, Graham Heap, Tariq Ahmad, Vincent Plagnol, Anthony W. Segal, Stephan Targan, Dan Turner, Paivi Saavalainen, Martti Farkkila, Kimmo Kontula, Aarno Palotie, Steven R. Brant, Richard H. Duerr, Mark S. Silverberg, John D. Rioux, Rinse K. Weersma, Andre Franke, Luke Jostins, Carl A. Anderson, Jeffrey C. Barrett, Daniel G. MacArthur, Chaim Jalas, Harry Sokol, Ramnik J. Xavier, Ann Pulver, Judy H. Cho, Dermot P. B. McGovern, Mark J. Daly

**Affiliations:** 1 Medical and Population Genetics, Broad Institute, Cambridge, MA, United States of America; 2 Department of Biomedical Data Science, Stanford University, Stanford, CA, United States of America; 3 Analytical and Translational Genetics Unit, Massachusetts General Hospital, Boston, MA, United States of America; 4 Institute for Molecular Medicine Finland (FIMM), University of Helsinki, Helsinki, Finland; 5 Department of Mathematics and Statistics, University of Helsinki, Helsinki, Finland; 6 Translational Genomics Unit, F. Widjaja Foundation Inflammatory Bowel and Immunobiology Research Institute, Cedars-Sinai Medical Center, Los Angeles, CA, United States of America; 7 Hadassah-Hebrew University Medical Center, Endocrinology and Metabolism Service Department of Internal Medicine, Jerusalem, Israel; 8 Department of Genetics and Genomic Sciences, Icahn School of Medicine at Mount Sinai, New York, NY, United States of America; 9 Department of Genetics and Medicine, Albert Einstein College of Medicine, Bronx, NY, United States of America; 10 Faculty of Natural Sciences, University of Haifa, Haifa, Israel; 11 Division of Medicine, University College London, London, United Kingdom; 12 UCL Genetics Institute, University College London, London, United Kingdom; 13 Institute of Clinical Molecular Biology, Christian-Albrechts-University of Kiel, Kiel, Germany; 14 Gastroenterology Department, Saint-Antoine Hospital, AP-HP, UPMC Univ Paris, Paris, France; 15 Department of Internal Medicine, University Hospital Schleswig-Holstein, Kiel, Germany; 16 Gastroenterology Practice, Minden, Germany; 17 IBD Pharmacogenetics, Royal Devon and Exeter NHS Trust, Exeter, United Kingdom; 18 Peninsula College of Medicine and Dentistry, Exeter, United Kingdom; 19 Juliet Keidan Institute of Pediatric Gastroenterology and Nutrition, Shaare Zedek Medical Center, The Hebrew University of Jerusalem, Jerusalem, Israel; 20 Research Programs Unit, Immunobiology, and Department of Medical and Clinical Genetics, University of Helsinki, Helsinki, Finland; 21 Department of Medicine, Division of Gastroenterology, Helsinki University Hospital, Helsinki, Finland; 22 Department of Medicine, University of Helsinki and Helsinki University Central Hospital, Helsinki, Finland; 23 Department of Neurology, Massachusetts General Hospital, Boston, MA, United States of America; 24 Meyerhoff Inflammatory Bowel Disease Center, Department of Medicine, School of Medicine, Johns Hopkins University, Baltimore, MD, United States of America; 25 Department of Epidemiology, Bloomberg School of Public Health, Johns Hopkins University, Baltimore, MD, United States of America; 26 Division of Gastroenterology, Hepatology and Nutrition, Department of Medicine, University of Pittsburgh School of Medicine, Pittsburgh, PA, United States of America; 27 Department of Human Genetics, University of Pittsburgh Graduate School of Public Health, Pittsburgh, PA, United States of America; 28 Inflammatory Bowel Disease Centre, Mount Sinai Hospital, Toronto, Ontario, Canada; 29 Research Center, Montreal Heart Institute, Montréal, Québec, Canada; 30 Department of Medicine, Université de Montréal, Montréal, Québec, Canada; 31 Department of Gastroenterology and Hepatology, University Medical Center Groningen, Groningen, The Netherlands; 32 Wellcome Trust Centre for Human Genetics, Oxford University, Oxford, United Kingdom; 33 Wellcome Trust Sanger Institute, Wellcome Trust Genome Campus, Hinxton, United Kingdom; 34 Bonei Olam, Center for Rare Jewish Genetic Disorders, Brooklyn, NY, United States of America; 35 Gastrointestinal Unit and Center for the Study of Inflammatory Bowel Disease and Center for Computational and Integrative Biology, Massachusetts General Hospital, Harvard Medical School, Boston, MA, United States of America; 36 Department of Psychiatry and Behavioral Sciences, Johns Hopkins University School of Medicine, Baltimore, MD, United States of America; 37 Icahn School of Medicine at Mount Sinai, Dr Henry D. Janowitz Division of Gastroenterology, New York, NY, United States of America; Case Western Reserve University School of Medicine, UNITED STATES

## Abstract

As part of a broader collaborative network of exome sequencing studies, we developed a jointly called data set of 5,685 Ashkenazi Jewish exomes. We make publicly available a resource of site and allele frequencies, which should serve as a reference for medical genetics in the Ashkenazim (hosted in part at https://ibd.broadinstitute.org, also available in gnomAD at http://gnomad.broadinstitute.org). We estimate that 34% of protein-coding alleles present in the Ashkenazi Jewish population at frequencies greater than 0.2% are significantly more frequent (mean 15-fold) than their maximum frequency observed in other reference populations. Arising via a well-described founder effect approximately 30 generations ago, this catalog of enriched alleles can contribute to differences in genetic risk and overall prevalence of diseases between populations. As validation we document 148 AJ enriched protein-altering alleles that overlap with "pathogenic" ClinVar alleles (table available at https://github.com/macarthur-lab/clinvar/blob/master/output/clinvar.tsv), including those that account for 10–100 fold differences in prevalence between AJ and non-AJ populations of some rare diseases, especially recessive conditions, including Gaucher disease (*GBA*, p.Asn409Ser, 8-fold enrichment); Canavan disease (*ASPA*, p.Glu285Ala, 12-fold enrichment); and Tay-Sachs disease (*HEXA*, c.1421+1G>C, 27-fold enrichment; p.Tyr427IlefsTer5, 12-fold enrichment). We next sought to use this catalog, of well-established relevance to Mendelian disease, to explore Crohn's disease, a common disease with an estimated two to four-fold excess prevalence in AJ. We specifically attempt to evaluate whether strong acting rare alleles, particularly protein-truncating or otherwise large effect-size alleles, enriched by the same founder-effect, contribute excess genetic risk to Crohn's disease in AJ, and find that ten rare genetic risk factors in *NOD2* and *LRRK2* are enriched in AJ (p < 0.005), including several novel contributing alleles, show evidence of association to CD. Independently, we find that genomewide common variant risk defined by GWAS shows a strong difference between AJ and non-AJ European control population samples (0.97 s.d. higher, p<10^−16^). Taken together, the results suggest coordinated selection in AJ population for higher CD risk alleles in general. The results and approach illustrate the value of exome sequencing data in case-control studies along with reference data sets like ExAC (sites VCF available via FTP at ftp.broadinstitute.org/pub/ExAC_release/release0.3/) to pinpoint genetic variation that contributes to variable disease predisposition across populations.

## Author summary

The Ashkenazim are a people with ancestry in northern-European Jewish groups. A founder effect caused a bottleneck in this population approximately one thousand years ago, resulting in a group of enriched alleles in their genetic makeup. A higher documented prevalence of Crohn’s Disease in the Ashkenazim indicates that some enriched alleles may confer risk of having this disease. By studying which genes are enriched, and which of these contribute to Crohn’s Disease risk, we are better able to understand the genetic architecture of the affected population, and of the disease itself. Further, we are able to develop a resource containing tables of significantly enriched alleles that are known or suspected to contribute to other disease.

## Introduction

Genetic population isolates like the Ashkenazim, Jews who trace their ancestry to eleventh century central European Jewish groups[1], have previously facilitated the mapping of alleles contributing to human disease predisposition[2–5]. The documented 2–4 fold enrichment of Crohn’s Disease (CD) prevalence in the Ashkenazi Jewish (AJ) population[6,7] motivated the use of exome sequencing and genome-wide array data to evaluate the degree to which bottleneck-enriched protein-altering alleles and unequivocally implicated common variants contribute an excess CD genetic risk to AJ[6]. Despite the progress in mapping genes and alleles for rare diseases with increased prevalence in the AJ population, precise estimates of the risk-allele frequency and the carrier rate in the AJ population have not yet been determined[8]. Through this study, we provide a frequency resource of protein-coding alleles from over 2,000 non-CD AJ individuals with low admixture that will serve to improve interpretation of rare disease risk alleles in the AJ population and which we employ to discover new Crohn’s risk alleles by comparison to 1855 AJ Crohn’s cases.

## Results

We generated a jointly called whole-exome sequence dataset consisting of 18,745 individuals from international Inflammatory Bowel Disease (IBD) and non-IBD cohorts[9,10] (S1 Fig). Given the increased prevalence of Crohn’s disease in the AJ population, our global sequencing efforts had specifically included 5,652 individuals self-reporting as Jewish and, as we aimed to focus on variation observed in the AJ population in comparison to reference populations in ExAC[9,11] (including non-Finnish Europeans (NFE), Latino (AMR), and African/African-American (AFR)) populations, we chose a model-based approach to estimate the ancestry of the study population using ADMIXTURE[12].

To identify AJ individuals and estimate admixture fractions we used a set (n = 21,066) of LD-pruned common variants (MAF>1%, see Supplementary Note for additional details) filtered for genotype quality (GQ>20). The 18,745 individuals were assigned to four groups (K = 4) using ADMIXTURE (further described in Supplementary Note, also see S3 Fig). One group of 5,685 individuals was found consisting mostly (84%) of self-reported AJ individuals, while 3,522 of these individuals were further found with high ancestry fraction (> 0.9) mapping to this group (S2 Fig, S1 Table). Thus, many self-reported AJ individuals were not included, as they did not have high enough ascertained AJ ancestry fraction. As we were interested in computing an enrichment statistic that would not be affected by possible admixture, we obtained alternate (non-reference) allele frequency estimates by restricting the enrichment analysis to the 2,178 non-IBD Ashkenazi Jewish individuals that passed QC and relatedness filtering and had AJ ancestry fraction (genotype ancestry grouping closely with other AJ individuals) of > 0.9. Our study includes exomes throughout Europe and Israel but the vast majority (86%) of these high ancestry fraction AJ individuals were collected in major US cities including Los Angeles, Boston, Baltimore, and New York (S2 Table).

**Fig 1:**
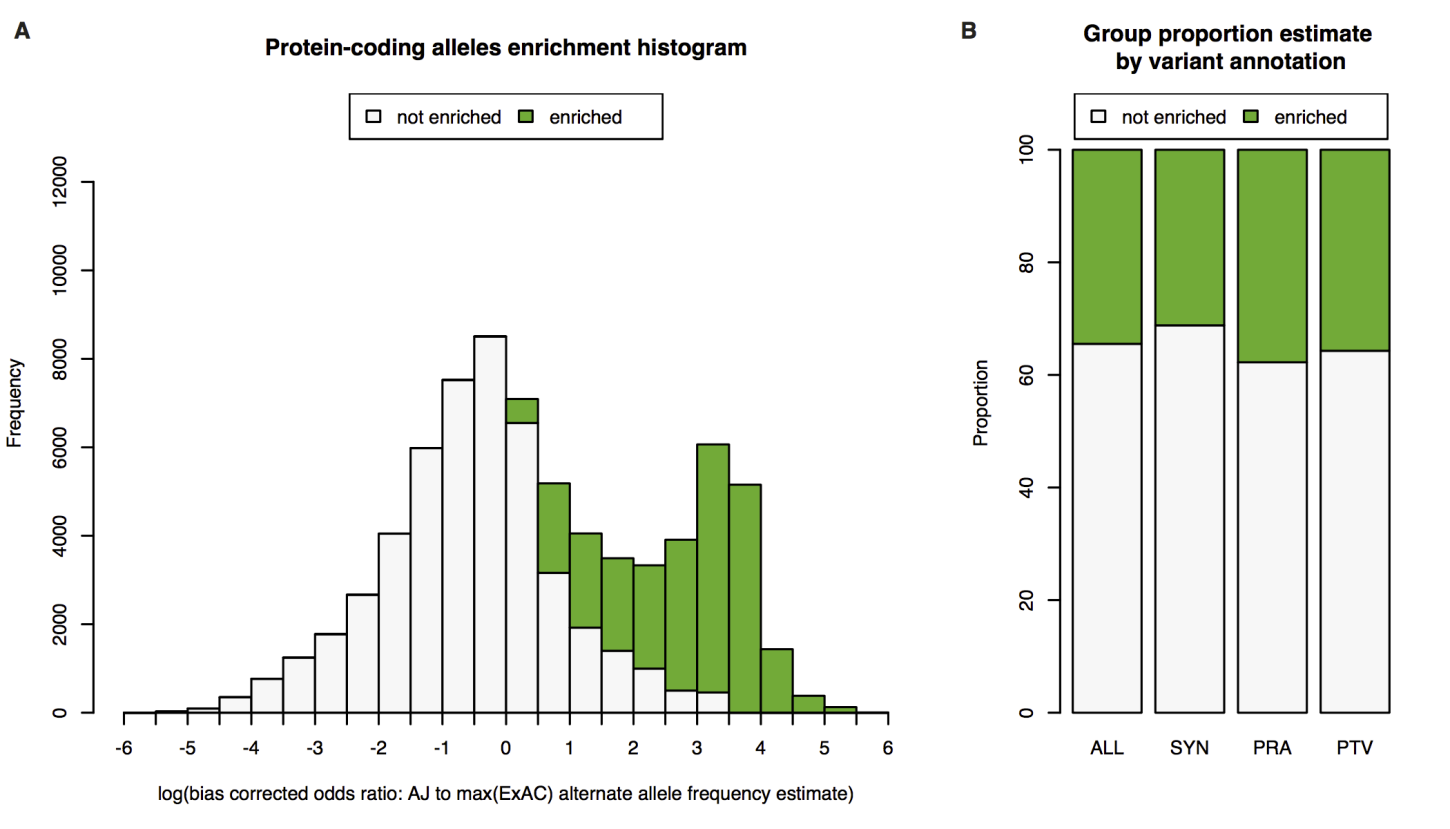
Enrichment of alleles discovered in AJ exome sequencing project. **A)** Histogram of estimated log enrichment statistic, defined as the log of the bias corrected odds ratio comparing the allele frequency in AJ population to the maximum allele frequency estimated from NFE, AFR, and AMR populations in ExAC. For each histogram bin we show a bar plot of the expected number of alleles belonging to the two groups we analyzed: 1) enriched (green) and 2) not enriched (white). **B)** Bar plots of estimated percentage of alleles belonging to the two groups we analyzed for all protein-coding (ALL), synonymous (SYN), protein-altering (PRA), and protein-truncating variants (PTV). An estimate of 34% of protein-coding alleles observed in AJ have a mean shift of 15-fold increased odds of the alternate allele compared to other reference populations. This observation is supported by the property that compared to intergenic variants, coding variants tend to be younger for a given frequency and the more pathogenic a variant, the younger it is, therefore tending to be population specific[13].

To explore AJ exome population genetics, including proportion of enriched alleles and degree of enrichment, we used the observed alternate allele counts and total number of alleles available from ExAC release 0.3 dataset [n_total_ = 60,706; NFE (n = 31,902; after excluding AJ individuals from ExAC), AFR (n = 5,203), and AMR (n = 5,789)]. We focused on protein-coding alleles with estimated allele frequency of at least 0.002 and less than .1 in AJ (n_alleles_ = 73,228; practical cutoff of what could be statistically defined as convincing enrichment, see S4 Fig), and applied a one-sided Fisher’s exact test on allele counts (see Supplementary Note), to classify the observed alleles into two groups: “enriched” or “not enriched”. This analysis identified 34% of protein-coding alleles as significantly enriched, with mean 15-fold increased odds of the alternate allele compared to other populations. Different proportions of alleles belong to the enriched group depending on variant annotation: 36% for predicted protein-truncating variants (PTV); 38% for predicted protein-altering variants (PRA); and 31% for synonymous variants. The substantially higher PTV+PRA:synonymous ratio observed in the enriched category is consistent with those alleles being drawn randomly from a large pool of much rarer alleles (where the functional:synonymous ratio is higher[3]) and abruptly boosted in frequency (Fig 1, p < 10^−16^ across comparisons of PTV and PRA to synonymous variants, two-proportion test, Supplementary Note). Since much rarer alleles have a higher probability of being damaging (e.g., they have a higher missense/synonymous ratio), the advantage to gene mapping arises from the fact that enriched alleles of a certain frequency are more damaging/deleterious on average than non-enriched alleles of the same frequency.

Additionally, we may expect that a “depleted” set of alleles arises from the founder effect, but in reality, many of these already rare variants are simply eliminated during the bottleneck. Of course, it is more difficult and less interesting to search for depleted alleles, as their absence provides no opportunity to obtain significant statistics on population enrichment or disease association.

We intersected the list of protein-coding alleles identified in the AJ exome sequencing study with alleles reported to be pathogenic with no conflicting evidence (n = 42,226) in ClinVar[14] resulting in 148 alleles found both in ClinVar and with p-value less than .005 of belonging to the AJ enriched set (S1 Data File). In OMIM, 48 of the 148 alleles included documentation of a disease subject with AJ ancestry (Table 1). This set of enriched alleles includes all of the major AJ mutations for 8 diseases described in the American College of Medical Genetics and Genomics 2008 screening guideline study[15]. In the setting of autosomal recessive disorders these differences in population allele frequencies may contribute a factor proportional to the squared enrichment difference to genetic risk and prevalence between populations (see Supplementary Note). For instance, a 19-fold enriched frameshift indel, p.Tyr427IlefsTer5, in *HEXA*, contributes a 361-fold enrichment in genetic risk in AJ to non-AJ population to Tay-Sachs disease. Enrichment in this large adult Ashkenazi exome database reinforces recent publications of founder mutations for rare pediatric disorders including *FKTN* (Walker Warburg syndrome)[16], *CCDC65* (Primary ciliary dyskinesia)[17], *TMEM216* (Joubert syndrome)[18], *C11orf73* (Leukoencephalopathy)[19]; *PEX2* (Zellweger syndrome)[20], *VPS11* (Hypomyelination and developmental delay)[21] and *BBS2* (Bardet-Biedl syndrome)[22]. While many alleles on this pathogenic list may demonstrate incomplete penetrance (as in the case of p.V726A in *MEFV*[23] for Familial Mediterranean fever) and some may not show recessive inheritance, this resource should provide considerable assistance in gene discovery and clinical genetic screening in AJ (S2 Data File).

**Table 1:** Forty-eight ClinVar “pathogenic” alleles enriched in AJ. HGVS and Gene is the allele nomenclature in ClinVar and gene symbol, respectively. Enrichment odds ratio corresponds to the bias corrected comparison of allele frequency in AJ (AJ AF) to maximum frequency among three population groups (max EXAC AF): 1) NFE; 2) AMR; and 3) AFR. Curated trait is based on the trait description in the Online Mendelian Inheritance in Man (OMIM) and is independent of effect size as a Crohn’s risk allele. Inheritance corresponds to the inheritance description in OMIM (AR: autosomal recessive, AD: autosomal dominant, risk factor: not specified genetic risk factor). Alleles are sorted in decreasing order by AJ AF.

Variant	HGVS	Gene	Enrichment Odds Ratio	AJ AF	Max ExAC AF	Curated Traits	Inheritance
**16:3293310:A:G**	p.Val726Ala	*MEFV*	26.08	0.0416	0.0017	Familial Mediterranean fever	AR
**5:150723155:C:A**	p.Gly87Val	*SLC36A2*	3.51	0.0414	0.0122	Hyperglycinuria	AD
**1:155205634:T:C**	p.Asn409Ser	*GBA*	11.16	0.0296	0.0027	Susceptibility to Lewy bod dementia, Gaucher’s disease, Susceptibility to late onset Parkinson’s disease	AR
**4:187201412:T:C**	p.Phe301Leu	*F11*	47.17	0.0273	0.0006	Hereditary factor XI deficiency	AR
**13:20763553:CA:C**	p.Leu56Argfs	*GJB2*	39.19	0.0199	0.0005	Autosomal recessive deafness	AR
**4:187195347:G:T**	p.Glu135Ter	*F11*	28.20	0.0195	0.0007	Factor XI deficiency	AR
**12:14421038:G:A**	p.Arg49Cys	*PRB3*	16.12	0.0189	0.0012	Salivary peroxidase	AR
**9:111662096:A:G**	c.2204+6T>C	*IKBKAP*	45.22	0.0168	0.0004	Familial dysautonomia	AR
**15:72638920:G:GGATA**	p.Tyr427IlefsTer5	*HEXA*	19.14	0.0122	0.00064	Tay-Sachs disease	AR
**1:125848678:C:T**	p.Arg4192His	*USH2A*	13.63	0.0106	0.0008	Retinitis pigmentosa	AR
**22:29091207:G:A**	p.Ser428Phe	*CHEX2*	50.06	0.0103	0.0002	Hereditary cancer, multiple types	Risk factor
**10:99371368:TGAG:T**	p.Glu315del	*HOGA1*	29.28	0.0101	0.0003	Primary hyperoxaluria	AR
**7:117282620:G:A**	p.Trp1282Ter	*CFTR*	23.64	0.0085	0.0004	Cystic fibrosis	AR
**11:17418602:C:T**	c.3992-9G>A	*ABCC8*	40.62	0.0076	0.0002	Hyperinsulinemic hypoglycemia	AR, AD
**17:3402294:A:C**	p.Glu285Ala	*ASPA*	40.36	0.0076	0.0002	Canavan disease	AR
**2:98986540:G:A**	c.101+1G>A	*CNGA3*	26.11	0.0074	0.0003	Achromatopsia	AR
**13:32914437:GT:G**	p.Ser1982Argfs	*BRCA2*	27.57	0.0069	0.0003	Hereditary cancer, multiple types	Risk factor
**9:97934315:T:A**	c.456+4A>T	*FANCC*	42.75	0.0069	0.0002	Fanconi anemia	AR
**9:108382330:G:GA**	p.Phe390Ilefs	*FKTN*	32.62	0.0067	0.0002	Limb-girdle muscular dystrophy-dystroglycanopathy	AR
**12:40734202:G:A**	p.Gly2019Ser	*LRRK2*	20.64	0.0064	0.0003	Parkinson’s disease	Risk factor
**17:41055964:C:T**	p.Arg83Cys	*G6PC*	11.04	0.0062	0.0006	Glycogen storage disease	AR
**1:26764719:A:G**	p.Lys42Glu	*DHDDS*	64.83	0.0051	0.0001	Retinitis pigmentosa	AR
**3:150690352:A:C**	p.Asn48Lys	*CLRN1*	46.26	0.0051	0.0001	Usher syndrome	AR
**12:49312533:GTA:G**	p.Ile293Profs	*CCDC65*	25.75	0.0048	0.0002	Ciliary dyskinesia without situs inversus	AR
**6:80878662:G:C**	p.Arg183Pro	*BCKDHB*	29.42	0.0046	0.0002	Maple syrup disease	AR
**10:56077147:G:A**	p.Arg245Ter	*PCDH15*	26.58	0.0046	0.0002	Usher syndrome	AR
**7:107555951:G:T**	p.Gly229Cys	*DLD*	26.55	0.0046	0.0002	Maple syrup disease	AR
**15:72638575:C:G**	c.1421+1G>C	*HEXA*	52.65	0.0044	0.0001	Tay-Sachs disease	AR
**15:72105913:G:A**	p.Arg311Gln	*NR2E3*	9.86	0.0042	0.0004	Enhanced s-cone syndrome	AR
**5:178699927:G:A**	p.Gln225Ter	*ADAMTS2*	129.41	0.0041	0.0000	Ehlers-Danlos syndrome, dermatosparaxis type	AR
**16:50745656:G:A**	p.Ala612Thr	*NOD2*	12.48	0.0039	0.0003	Early-onset sarcoidosis	Risk factor
**11:6415434:G:T**	p.Arg498Leu	*SMPD1*	41.53	0.0039	0.0001	Niemann-Pick disease	AR
**11:61161437:G:T**	p.Arg73Leu	*THEM216*	27.77	0.0039	0.0001	Joubert syndrome	AR
**1:53676583:CAG:C**	pLys414ThrfsTer7	*CPT2*	78.34	0.0037	0.0000	Carnitine palmitoyltransferase II deficiency	AR
**1:53676688:T:C**	p.Phe448Leu	*CPT2*	78.35	0.0037	0.0000	Carnitine palmitoyltransferase II deficiency	AR
**3:172737276:C:T**	p.Arg283Gln	*SPATA16*	9.79	0.0037	0.0004	Spermatogenic failure	AR
**11:86017416:G:C**	p.Val54Leu	*C11orf73*	47.03	0.0037	0.0001	Hypomyelinating leukodystrophy	AR
**8:77896070:G:A**	p.Arg119Ter	*PEX2*	20.03	0.0034	0.0002	Peroxisome biogenesis disorder	AR
**11:118951899:T:G**	p.Cys845Gly	*VPS11*	190.98	0.0030	0.0000	Hypomyelinating leukodystrophy	AR
**6:80203353:G:A**	p.Gln279Ter	*LCA5*	29.25	0.0028	0.0001	Leber congenital amaurosis	AR
**19:7591645:A:G**	c.406-2A>G	*MCOLN1*	21.93	0.0028	0.0001	Mucolipidosis	AR
**16:56530894:C:G**	p.Arg632Pro	*BBS2*	29.37	0.0028	0.0001	Retinitis pigmentosa	AR
**17:41276044:ACT:A**	p.Glu23Valfs	*BRCA1*	10.04	0.0025	0.0003	Hereditary cancer, multiple types	Risk factor
**4:100543913:G:T**	p.Gly865Ter	*MTTP*	40.38	0.0025	0.0001	Abetalipoproteinaemia	AR
**2:99013302:G:A**	p.Gly557Arg	*CNGA3*	29.36	0.0023	0.0001	Achromatopsia	AR
**7:107557794:G:A**	p.Glu375Lys	*DLD*	26.44	0.0021	0.0001	Maple syrup disease	AR
**17:41209079:T:TG**	p.Gln1756Profs	*BRCA1*	8.80	0.0021	0.0002	Hereditary cancer, multiple types	Risk factor
**10:99371292:G:T**	p.Gly287Val	*HOGA1*	22.01	0.0021	0.0001	Primary hyperoxaluria	AR

To assess whether AJ-enriched protein-coding alleles also contribute to the established difference in CD genetic risk we performed case-control association analyses. Since individuals with only partial AJ ancestry will still carry bottleneck-enriched alleles, here we included samples with estimated AJ ancestry fraction > 0.4 (Supplementary Note, S2 Fig), resulting in a dataset of 4,899 AJ samples (1,855 Crohn’s disease and 3,044 non-IBD). To improve ability to detect a true association, we performed a meta-analysis with CD and non-IBD case-control exome sequencing data from two additional ancestry groups: 1) non-Finnish European (NFE) (2,296 CD and 2,770 non-IBD); and 2) Finnish (FIN) (210 CD and 9,930 non-IBD samples) from a separate callset described in a previous publication[24] for a total of 4,361 CD samples and 15,744 non-IBD samples. By calling additional non-AJ samples, we hoped to discern which of the AJ-enriched alleles contributed a significant risk factor across all populations. The meta-analysis performed across several populations described should mitigate biases by confirming consistency in effect size across these population groups.

**Fig 2:**
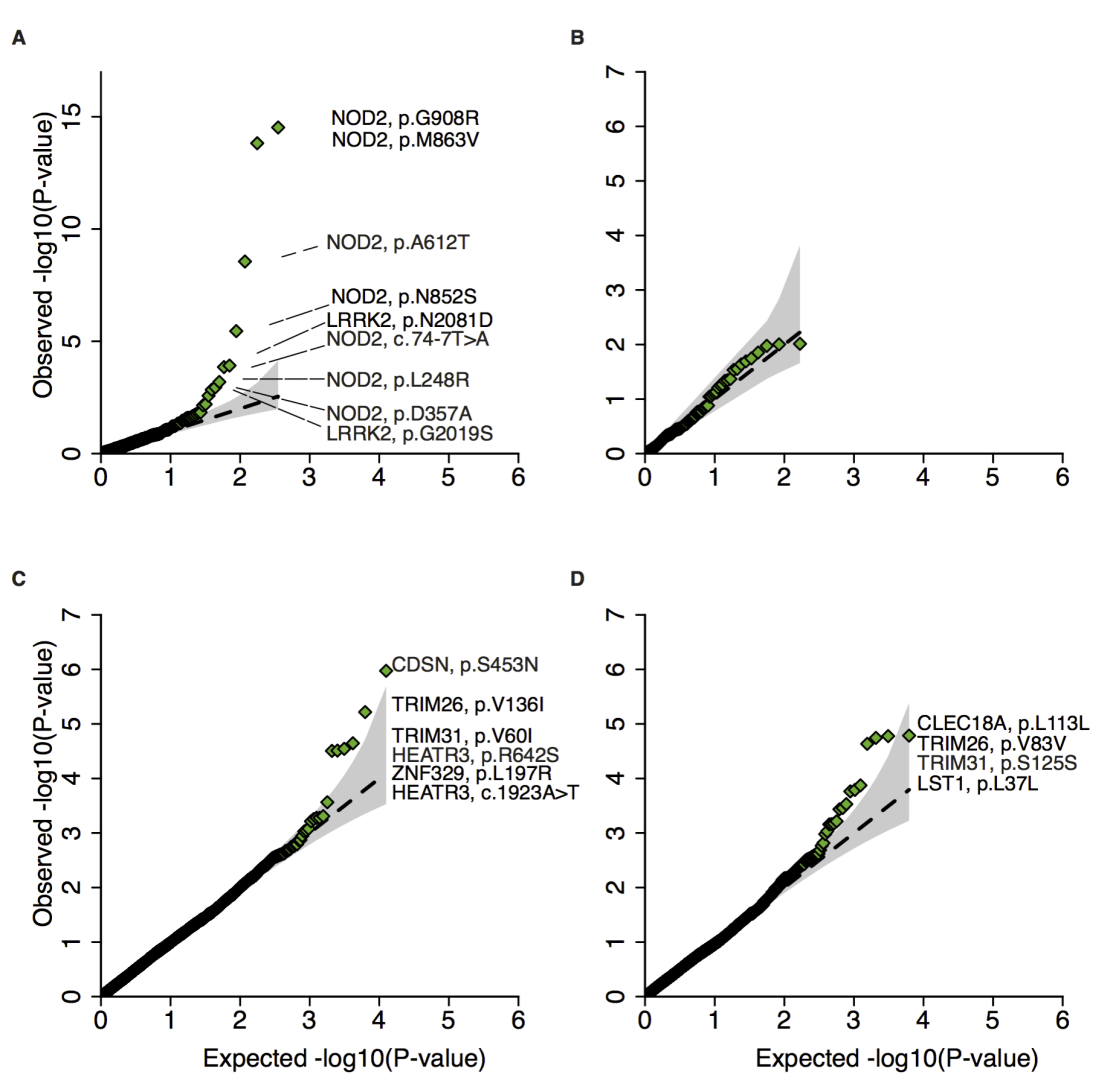
Q-Q plots of enriched alleles. Q-Q plots of Crohn’s disease association for AJ enriched **A**) protein-altering (protein-truncating and missense) and **B**) synonymous alleles in GWAS regions; and AJ enriched **C**) protein-altering and **D**) synonymous alleles outside of GWAS regions. For each Q-Q plot variants with a corresponding p-value less than or equal to a threshold where expected number of false discoveries is equal to one are annotated. The black dashed line is y = x, and the grey shapes show 95% confidence interval under the null.

Study-specific association analysis was performed with Firth bias-corrected logistic regression[25,26] and four principal components as covariates using the software package EPACTS[27] (S5 Fig). We combined association statistics in a meta-analysis framework using the Bayesian models in Band et al.[28]. We used the correlated effects model, obtained a Bayes factor (BF) by comparing it with the null model where all the prior weight is on an effect size of zero, reported p-value approximation using the BF as a test statistic, and assessed whether heterogeneity of effects exist across studies for downstream QC (see Supplementary Note). We separately assessed CD associations of enriched protein-altering (PRA) and synonymous (SYN) alleles in protein-coding genes in CD implicated GWAS loci (n_gwas,pra_ = 351; n_gwas,syn_ = 167), and outside implicated GWAS loci (n_non-gwas,pra_ = 12,529; n_non-gwas,syn_ = 6,202, Fig 2). See Methods and Materials for a description of these loci.

We identified ten AJ enriched CD risk alleles (p<0.005): the previously published risk haplotypes in *LRRK2* and *NOD2* (*LRRK2*: p.N2081D; *NOD2*: p.N852S, p.G908R, p.M863V+p.fs1007insC)[29,30], in addition to newly implicated alleles (*NOD2*: p.A612T, p = 2.8x10^-9^; c.74-7T>A, p = 1.4x10^-4^; p.L248R, p = 6.4x10^-4^; p.D357A, p = 0.0011; *LRRK2*: p.G2019S, p = 0.0014, a Parkinson’s disease risk allele[31]). To assess whether the new *NOD2* enriched alleles are conditionally independent of the previously established associated *NOD2* alleles we performed conditional haplotype association analysis in PLINK and Bayesian model averaging[32] for variable selection, both of which suggested independent effects for all alleles (S6 Fig, S3 Table).

Deviation from additivity can contribute additionally to individual risk but has been difficult to document in complex disease associations with modest ORs. Despite the functional relationship between *LRRK2* and *NOD2*[33], we do not observe deviation from additivity between *LRRK2* and *NOD2* (p = 0.273); that is, the effect of mutations in both *LRRK2* and *NOD2* is no greater than the sum of their individual effects. We assessed whether composite risk carriers (carrier of more than one variant allele) had evidence of deviation from additivity. Deviation from additivity has been reported for p.fs1007insC, p.G908R, and p.R702W in *NOD2*[34,35]. In our AJ exome sequencing data set we estimate a 1-hit effect equal to 1.82 (95% confidence interval [1.59, 2.07]) and a 2-hit effect equal to 8.24 (95% confidence interval [6.06, 11.21]; we found similar evidence for departure from additivity when restricting the analysis to the newly reported alleles only: p = 0.00357, odds ratio = 7.53). We confirmed this finding using the larger non-AJ Crohn’s disease ImmunoChip dataset to provide a more precise estimate of the 1-hit effect (OR = 2.17; 95% confidence interval [2.07, 2.27], S4 Table) and the non-additive 2-hit effects in *NOD2* (OR = 9.93; 95% confidence interval [8.88, 11.13], S5 Table). We found no evidence of deviation from additivity for the associated protein-altering alleles in *LRRK2* (p = 0.418).

**Fig 3:**
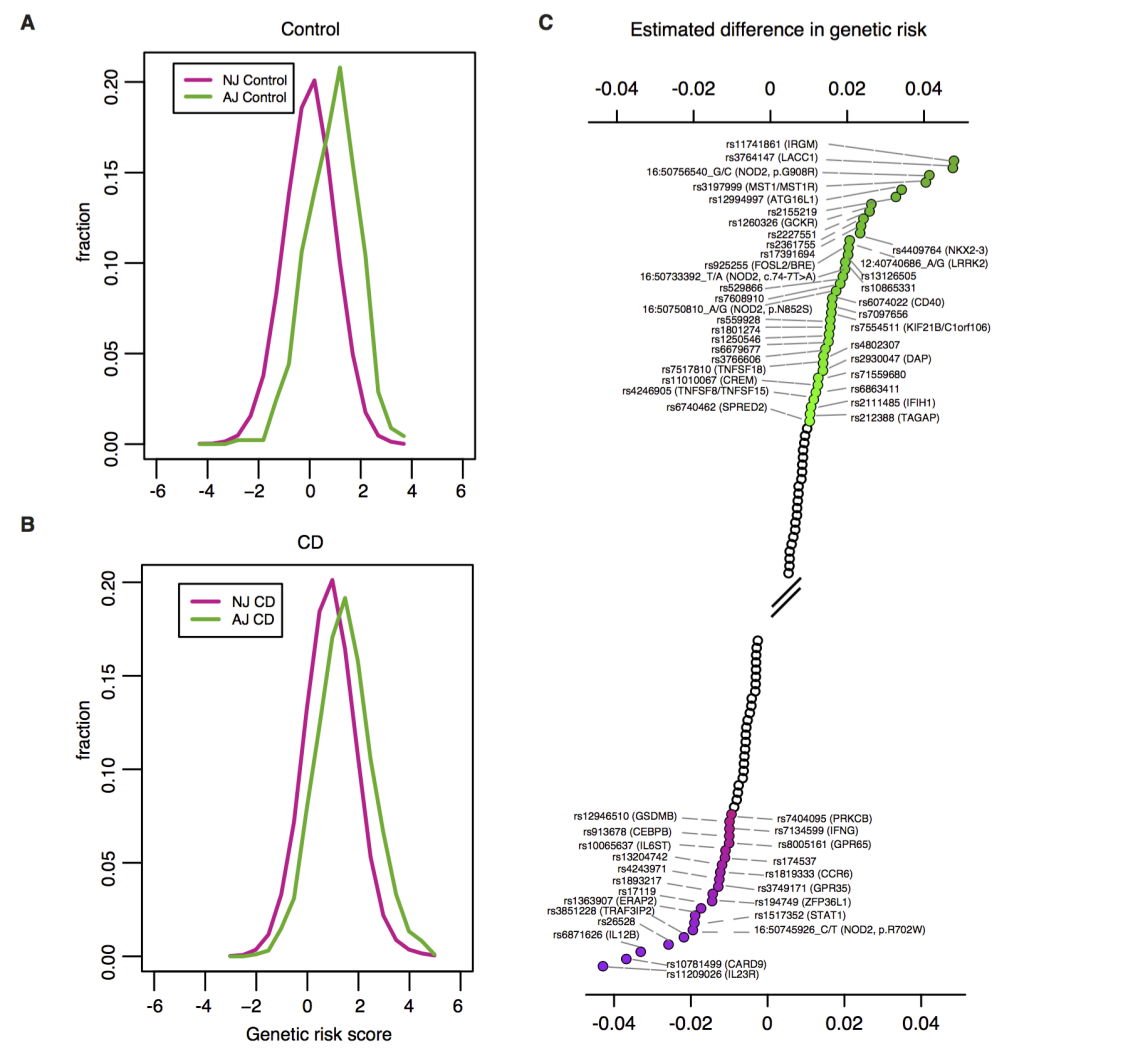
AJ individuals have higher CD polygenic risk score than NJ controls. NJ: non-Jewish; AJ: Ashkenazi Jewish; CD: Crohn’s disease; PRS: polygenic risk score. **A**) Density plot of CD polygenic risk scores in 454 AJ (green) and 35,007 NJ(purple)controls. AJ controls have higher CD polygenic risk score than NJ controls (0.97 s.d. higher, p<10^−16^). **B**) Density plot of CD polygenic risk scores in 1,938 AJ (green) and 20,652 NJ CD (purple) cases (0.54 s.d. higher, p<10^−16^). For both density plots the scores have been scaled to NJ controls, thus resulting in an NJ control PRS density of mean equal to 0 and variance equal to 1 (see Online Methods). **C**) Ranked (decreasing order) CD associated variants by estimated contribution to the differences in genetic risk between AJ and NJ. Associated variants with estimated contribution greater than or equal to 0.01, computed as 2 log(odds ratio) (AJ frequency—NJ frequency), assuming additive effects on the log scale, are highlighted in green. Associated variants with estimated contribution less than or equal to -0.01 are highlighted in purple. Forward slashes represent a break in variants highlighted.

Given that enriched genetic variants in *NOD2* and *LRRK2* contribute to differences in CD risk in AJ population, we next asked whether unequivocally established common variant associations contribute to differences in CD genetic risk. We performed polygenic risk score (PRS) analysis using reported effect size estimates from 124 CD alleles including those reported in a previously published study[36] and four variants in *IL23R* from a recent fine-mapping study[37], and excluding variants in *NOD2* and *LRRK2*. We observed an elevated PRS for AJ compared to non-Jewish controls (0.97 s.d. higher, p<10^−16^; Fig 3A; number of non-AJ controls = 35,007; number of AJ controls = 454), and as expected when performing the PRS analysis using OR calculated from non-Jewish subset of iCHIP data the signal still remains (p<10^−16^, S7 Fig). We observed a similar trend for the CD samples (0.54 s.d. higher; p<10^−16^; Fig 3B; number of non-AJ CD cases = 20,652; number of AJ CD cases = 1,938). We demonstrate this is not a systematic property of common risk alleles in AJ by running the same comparison using instead the comparable set of established schizophrenia associated alleles from the Psychiatric Genomics Consortium[38].

To quantify the relative contribution of CD-implicated alleles to the difference in genetic risk between AJ and non-AJ populations we estimated the expected PRS value of an individual and expected difference in PRS between two populations by simply using summary statistics including the frequency of the minor allele in the two populations and the corresponding odds ratio (Supplementary note, S6 Fig).

We applied the approach to all CD implicated alleles and observed that variants in GWAS loci annotated as *IRGM*, *LACC1*, *NOD2*, *MST1*, *ATG16L1*, *GCKR*, *NKX2-3*, and *LRRK2*[36] contribute substantially (>0.01) to the increased genetic risk observed in AJ. It is possibly relevant that variants contributing to increased risk in AJ include many autophagy/intracellular defense genes (*IRGM*, *ATG16L1*, *LRRK2*), while those contributing to increased risk in non-AJ include many anti-fungal/Th17/ILC3 genes[39] (*IL23R*, *IL12B*, *CARD9*, *TRAF3IP2*, *IL6ST*, *CEBPB*; Fig 3C).

Both documented variability in the occurrence of CD over time[40,41] and substantial uncertainty in reported CD prevalence estimates[42,43] impact our ability to precisely estimate the overall contribution of genetics to the established difference in prevalence between populations. To interpret the impact of shifts in genetic risk score on differences in prevalence, we used the logit risk model[35] and evaluated a new estimate of disease probability, p_new_, assuming an initial disease probability, p_0_, and multiple values for the differences in genetic risk.

Assuming log-additive effects, and a logit-risk model, we estimate that the observed differences in genetic risk between the AJ and non-AJ populations contribute an expected 1.5-fold increase in disease prevalence in a population with environmental risk factors corresponding to AJ and baseline genetic risk corresponding to non-AJ populations (S7–S9 Figs). To address the extent to which non-additive effects in *NOD2* may impact the observed prevalence we assumed 1-hit and 2-hit odds ratios of 2.17 and 9.93, respectively. We attribute a 6.8% difference in the ratio of estimated disease prevalence in the AJ population to the deviation from additivity, suggesting a small effect on differences in population prevalence (Supplementary Note).

## Discussion

Analyzing data from 5,685 Ashkenazi Jewish exomes, we provide a systematic analysis of AJ enriched protein-coding alleles, which may contribute to differences in genetic risk to CD as well as numerous rare diseases, many of which are transmitted via autosomal recessive inheritance. We identified protein-altering alleles in *NOD2* and *LRRK2* that are conditionally independent and contribute to the excess burden of CD in AJ. We found evidence that common variant risk defined by GWAS shows a strong elevated difference between AJ and non-AJ European population samples (0.97 s.d. higher in controls, 0.54 s.d. higher in cases, p<10^−16^ in both), independent of *NOD2* and *LRRK2*[44]. Highly polygenic diseases are unlikely to have substantially altered incidence as a result of a bottleneck alone—for every enriched variant there are those depleted or lost entirely and population genetics simulations[45] suggest no systematic alteration of overall genetic burden as a function of a bottleneck. Thus, the strong (approximately 1.5-fold, see supplementary note) difference in Crohn’s incidence in concert with a systematic enrichment of risk-increasing alleles, unlikely to have arisen by chance, suggests non-random selection in the AJ population for higher CD risk alleles. It seems plausible that, rather than ‘selection for Crohn's' per se, this likely suggests a subset of Crohn's risk alleles may contribute to a common biological process (e.g., a specific immune response) or phenotype that was positively selected for in AJ[46–48]. Such weak, widespread ‘polygenic selection’ has previously been observed with respect to height-associated SNPs in Europe[49], where drift alone could not explain the systematic enrichment of height-increasing alleles in populations of Northern Europe vs. Southern Europe. We found that CD risk alleles that are systematically elevated in AJ are not unusually elevated in another well-established founder population for which we have extensive genotype data (Finland). In Finns, Crohn’s risk alleles were not systematically enriched—they were if anything slightly depleted with 69 risk alleles at higher frequency in Finns than NFE and 79 risk alleles at lower frequency in Finns than NFE. We also demonstrate this is not a systematic property of common risk alleles in AJ by running the same comparison using instead the comparable set of established schizophrenia associated alleles from the Psychiatric Genetics Consortium[50]. We mapped 102 schizophrenia-associated index SNPs to AJ frequency data and again observed no uneven distribution where risk alleles are systematically more or less common. In total, 52 risk alleles were at higher frequency in AJ than NFE and 50 risk alleles were higher frequency in NFE than AJ.

This study of CD in the AJ population confirms population-genetic expectations. First, recently bottlenecked populations are uniquely powered to discover alleles with markedly increases in frequency, and, as a consequence, contributors to differences in genetic risk across population groups. Second, while *NOD2* and published common variant associations contribute substantially to the genetic risk of CD, other genes with causal alleles that failed to pass through the bottleneck are missed, consistent with predictions from Zuk et al[4].

We provide an exome frequency resource of protein-coding alleles in AJ along with estimates of population-specific enrichment. The sets of enriched alleles should be carefully considered when performing case-control analysis. Population structure can easily lead to false positive associations, especially for low frequency and rare variants, if the AJ:nonAJ ratio is slightly different in cases and controls. Our approach and this resource will likely catalyze our understanding of the medical relevance of enriched alleles in population isolates. Most importantly, the frequency reference provides critical guidance in pinpointing or excluding specific risk factors in individuals in clinical and research settings.

## Materials and methods

### Initial variant call set

We generated a jointly called dataset consisting of 18,745 individuals from international IBD and non-IBD cohorts. Sequencing of these samples was done at Broad Institute.

### Ethics statement

All patients and control subjects provided informed consent. Recruitment protocols and consent forms were approved by Institutional Review Boards at each participating institutions (Protocol Title: The Broad Institute Study of Inflammatory Bowel Disease Genetics; Protocol Number: 2013P002634). All DNA samples and data in this study were denominalized.

### Cohort descriptions

For all cohorts, CD was diagnosed according to accepted clinical, endoscopic, radiological and histological findings.

### Target selection

G4L WES is a project specific product. It combines the Human WES (Standard Coverage) product with an Infinium Genome-Wide Association Study (GWAS) array. In addition to the array adding to the genomics data, it also acts as a concordance QC, linking 14 SNPs to the exome data. The processing of the exome includes Sample prep (Illumina Nextera), hybrid capture (Illumina Rapid Capture Enrichment - 37Mb target), sequencing (Illumina, HiSeq machines, 150bp paired reads), Identification QC check, and data storage (5 years). Our hybrid selection libraries typically meet or exceed 85% of targets at 20x, comparable to ~60x mean coverage. The array consists of a 24-sample Infinium array with ~245,000 fixed genome-wide markers, designed by the Broad. On average our genotyping call rates typically exceed 98%.

### Pre-processing

The sequence reads are first mapped using BWA MEM[51] to the GRCh37 reference to produce a file in SAM/BAM format sorted by coordinate. Duplicate reads are marked–these reads are not informative and are not used as additional evidence for or against a putative variant. Next, local realignment is performed around indels. This identifies the most consistent placement of the reads relative to potential indels in order to clean up artifacts introduced in the original mapping step. Finally, base quality scores are recalibrated in order to produce more accurate per-base estimates of error emitted by the sequencing machines.

### Variant discovery

Once the data has been pre-processed as described above, it is put through the variant discovery process, i.e. the identification of sites where the data displays variation relative to the reference genome, and calculation of genotypes for each sample at that site. The variant discovery process is decomposed into separate steps: variant calling (performed per-sample), joint genotyping (performed per-cohort) and variant filtering (also performed per-cohort). The first two steps are designed to maximize sensitivity, while the filtering step aims to deliver a level of specificity that can be customized for each project.

Variant calling is done by running Genome Analysis Toolkit’s (GATK) HaplotypeCaller in gVCF mode on each sample's BAM file(s) to create single-sample gVCFs. If there are more than a few hundred samples, batches of ~200 gVCFs are merged hierarchically into a single gVCF to make the next step more tractable. Joint genotyping is then performed on the gVCFs of all available samples together in order to create a set of raw SNP and indel calls. Finally, variant recalibration is performed in order to assign a well-calibrated probability to each variant call in a raw call set, and to apply filters that produce a subset of calls with the desired balance of specificity and sensitivity as described in Rivas et al. (2016)[24]. Samples with > = 10% contamination are excluded from call sets. Exome samples with less than 40% of targets at 20X coverage are excluded.

### Variant annotation

Variant annotation was performed using the Variant Effect Predictor (VEP) [cite PMID: 20562413] version 83 with Gencode v19 on GRCh37. Loss-of-function (LoF) variants were annotated using LOFTEE (Loss-Of-Function Transcript Effect Estimator, available at https://github.com/konradjk/loftee), a plugin to VEP. LOFTEE considers all stop-gained, splice-disrupting, and frameshift variants, and filters out many known false-positive modes, such as variants near the end of transcripts and in non-canonical splice sites, as described in the code documentation.

### Identification of Finnish samples

Finnish CD patients were recruited from Helsinki University Hospital and described in more detail previously[52,53]. We used the same exome sequencing dataset described in Rivas et al.[24]. We applied additional PC correction in the Finnish identified individuals to remove individuals with membership of Finnish sub-isolate (Northern Finland) and excluded based on PC2 0.015 (853 excluded, 826 controls, 27 IBD). We recalculated PCs and included the first four PCs in the association analysis.

### Identifying previously implicated GWAS loci

CD implicated GWAS loci were those loci defined as reaching genome-wide significance in International IBD Genetics Consortium studies (Jostins, Ripke et al., Nature 2012) and (Liu et al., Nature Genetics 2015)—Credible sets of SNPs around index associations were defined as in (Huang et al., Nature 2017) for fine-mapped loci, and for others credible sets were defined as all SNPs with r^2^ > 0.6 to the index variant. Genes within 50 kb of the span of credible set SNPs were considered “implicated’ by GWAS.

### Ancestry estimation and quality control

As the present study aimed to focus on variation observed in Ashkenazi Jewish (AJ) population in comparison to reference populations in ExAC including (non-Finnish Europeans (NFE), Latino (AMR), and African/African-American (AFR)) we chose a model-based approach to estimate the ancestry of the study population using ADMIXTURE[12]. To identify AJ individuals and estimate admixture proportions we included a set (n = 21,066) of LD-pruned common variants (MAF>1%) after filtering for genotype quality (GQ>20) using the PLINK LD-pruning algorithm, whose description is available at http://pngu.mgh.harvard.edu/~purcell/plink/summary.shtml#prune.

For the parameters, we selected a window size of 50 SNPs, a window shift of 5 SNPs at each step, and the variance inflation factor (VIF) threshold equal to 2.

The 18,745 samples were assigned to four groups (K = 4), as ancestry was defined as having a single estimated ancestry fraction ≥ 0.4, and remaining three fractions < 0.4 (S2 Fig). Individuals mostly representing African/African-American and East-Asian ancestry (1,267 and 569 individuals respectively) were discarded from downstream analysis, as well as the 983 admixed individuals with none of the ancestry fractions ≥ 0.4. Thus, a total of 6,093 individuals were considered of Ashkenazi Jewish (AJ) ancestry, while 9,833 were considered to represent Non-Finnish Europeans (NFE). After sample QC and relatedness check, 5,685 individuals of Ashkenazi Jewish and 7,240 of non-Finnish European ancestry were found with valid IBD case/control status (S1 Table). Individuals with Ulcerative Colitis and unspecified and Indeterminate Colitis were further excluded, resulting in 4,899 AJ and 5,066 NFE individuals.

Prior to enrichment and association analysis, 81 samples (of total 18,745) were also filtered due to possible contamination (heterozygous/homozygous ratio < 1), excess of singletons (n>2000), deletion/insertion ratio (>1.5) and mean genotype quality (<40). 275 samples were excluded for relatedness (>0.35 cut-off). Genotypes with low genotype quality (<20) were filtered, in addition to variants with low call rate (<80%) and allele balance deviating from 70:30 ratio for greater than 40% of heterozygous samples if at least 7 heterozygous samples were identified.

As we were interested in computing an enrichment statistic that would not be affected by possible admixture, we obtained alternate allele frequency estimates by restricting the enrichment analysis to the 2,178 non-IBD Ashkenazi Jewish samples that passed QC and relatedness filtering and had AJ focused ancestry fraction > 0.9 (S1 Fig). Principal Component Analysis (PCA) was done in each ancestry group using the 21,066 variants. Sample QC was done using the Hail software while PCA, differential missingness and sample relatedness analysis was done using PLINK[54]. Hail is an open-source software framework for scalably and flexibly analyzing large-scale genetic data sets (https://github.com/broadinstitute/hail). Allele balance was calculated using PLINK/SEQ (https://atgu.mgh.harvard.edu/plinkseq/).

### Estimating fold-enrichment in AJ population compared to reference populations in ExAC

#### Statistical methods: Fisher’s exact test

To estimate which alleles are enriched in AJ compared to alleles in reference population groups in ExAC we applied Fisher’s exact test one-sided alternative (“greater”).

Using the number of alternate and reference alleles observed in AJ non-IBD samples and in the population (NFE, AFR or AMR) with the highest frequency from ExAC we compute a bias corrected log odds ratio estimate, $\hat{\beta_{i}}$, and its standard error, $\hat{{SE}_{i}}$, for odds of the alternate allele as described in the Software DataPlot developed by the National Institute of Standards and Technology (http://www.itl.nist.gov/div898/software/dataplot/refman2/auxillar/logoddra.htm, and http://www.itl.nist.gov/div898/software/dataplot/refman2/auxillar/logodrse.htm)

$$\hat{\beta_{i}}=\log\left({OR}_{i}\right)=\log\left(\frac{\left[\left(0.5+{ALT}_{AJ})∙(0.5+{REF}_{ExAC}\right)\right]}{\left[\left(0.5+{REF}_{AJ})∙(0.5+{ALT}_{ExAC}\right)\right]}\right),\;and$$

$${\hat{{SE}_{i}}}^{2}=\frac{1}{0.5+{REF}_{AJ}}+\frac{1}{0.5+{REF}_{ExAC}}+\frac{1}{0.5+{ALT}_{AJ}}+\frac{1}{0.5+{ALT}_{ExAC}}.$$

Precisely, $\hat{\beta_{i}}$ is the estimate of the log of the odds ratio of finding the alternate allele in AJ vs in the ExAC population with the highest allele frequency.

We classified a variant as ‘enriched’ if p-value was less than .05/73,228, where 73,228 is the number of variants analyzed with minor allele frequency between .002 and .1.

To estimate allele enrichment in AJ compared to reference populations we used 2,178 non-IBD Ashkenazi Jewish samples, after sample and relatedness QC.

We calculated alternate allele frequencies for the Ashkenazi Jewish population and used allele frequency information for NFE (n = 31,902; after excluding AJ individuals from ExAC), AFR (n = 5,203), and AMR (n = 5,789) available from ExAC release 0.3 dataset (n_total_ = 60,706) and focused on alleles where allele frequency information was available for AJ and the reference populations. For the enrichment plot we focused on alleles with estimated frequency of at least 0.002 in AJ (n_alleles_ = 106,377) and with alleles observed with an estimated frequency of at least .0001 in the reference populations with depth of coverage of at least 20X in at least 80% of the samples in ExAC.

#### Overlap of enriched alleles with ClinVar

We harmonized the XML and TXT releases of the ClinVar database (April 11, 2016 data release)[14] into a single tab-delimited text file using scripts that we have released publicly (https://github.com/macarthur-lab/clinvar). Briefly, we normalized variants using a Python implementation of vt normalize[55] and de-duplicated to yield a dataset unique on chromosome, position, reference, and alternate allele. A variant was considered 'pathogenic' if it had at least one assertion of either Pathogenic or Likely Pathogenic for any phenotype. A variant was considered 'conflicted' if it had at least one assertion of Pathogenic or Likely Pathogenic, and at least one assertion of Benign or Likely Benign, each for any phenotype. By these criteria, ClinVar contained n = 42,226 identified as pathogenic and non-conflicted. Intersecting with our dataset revealed that 148 belonged to the AJ enriched group with p-value less than .005.

#### Assessing Crohn’s disease association of protein-coding variation that may contribute to difference in disease prevalence in AJ

We focused Crohn’s disease association analysis of protein-coding variant to alleles that may account for difference in disease prevalence in AJ population to reference populations. To do so we focused on alleles with high probability of belonging to the enriched group. We included all samples with ADMIXTURE estimated AJ ancestry fraction of at least 0.4 (we excluded any samples that had alternative ancestry fraction of at least .4 in any other group). Samples with Ulcerative Colitis (n = 700), unspecified and Indeterminate Colitis (n = 86) were excluded from subsequent analysis. This resulted in a dataset of 4,899 AJ samples (1,855 Crohn’s disease and 3,044 non-IBD).

Study-specific association analysis was performed with Firth bias-corrected logistic regression test[25,26] and four principal components as covariates using the software package EPACTS version 3.2.6[27]. Minimum minor allele count (≥1) and variant call rate (≥0.8) filters were used.

For meta-analysis we combined association statistics using the Bayesian models and frequentist properties proposed in Band et al[28], which is a normal approximation to the logistic regression likelihood suggested by Wakefield[56]. As the authors of Band et al. indicate, one way of thinking about the approach is that it uses the study-wise estimated log-odds ratio (beta) and its standard error as summary statistics of the data. For each model of association, we assume a prior on the log odds ratio which is normally distributed around zero with a standard deviation of 0.2. By changing the prior on the covariance (or correlation) in effect sizes between studies we can formally compare models where: 1) the effects are independent across studies, and 2) the effects are correlated equally between studies. The final report of results is based on the correlated effects model. To address potential differences in effect sizes for the reported associated variants, we assessed heterogeneity of effects and did not find evidence (log_10_BF > 2). For each model we can obtain a Bayes factor (BF) for association by comparing it with the null model where all the prior weight is on an effect size of zero. We report p-value approximation using the Bayes factor as a statistic for model 2 where the effects are correlated between studies.

Association statistics were combined based on association analysis across three study groups: 1) AJ (1,855 CD and 3,044 non-IBD samples); 2) NFE (2,296 CD and 2,770 non-IBD); and 3) Finnish (FINN) (210 CD and 9,930 non-IBD samples) for a total of 4,361 CD samples and 15,744 non-IBD samples.

#### Conditional haplotype based testing and variable selection for *NOD2* alleles

In the conditional haplotype-based testing (—chap) analysis we used PLINK v1.08p[54] and set a minimum haplotype frequency of .001 (—mhf). We used PLINKSEQ (https://atgu.mgh.harvard.edu/plinkseq/), an open-source C/C++ library for working with human genetic variation data, and the Python bindings implemented in pyPLINKSEQ to perform Bayesian Model Averaging (BMA). We applied BMA[32] using the R package ‘BMA’ (https://cran.r-project.org/web/packages/BMA/BMA.pdf).

#### Polygenic risk scores

The polygenic risk scores were calculated for the international inflammatory bowel diseases consortium European samples. Details of these samples including the QC procedures were described in previous publications[37]. We used reported effect size estimates from 124 CD alleles including those reported in a previously published study[36] and four variants in *IL23R* from a recent fine-mapping study[37], and excluding variants in *NOD2* and *LRRK2*. We used 454 AJ controls; 1,938 AJ CD; 35,007 non-Jewish controls and 20,652 non-Jewish CD samples. Polygenic risk scores were calculated using array genotype data as the sum of the log odds ratio of the variants associated with CD. Scores for missing genotypes were replaced by the imputed expected value using PLINK[54]. Variants in *NOD2* and *LRRK2* were excluded from the analysis to assess whether polygenic signal was independent of those genes.

Let *PRS*_*i*_ be the polygenic risk score of individual *i*, assuming additive effects on the log-odds scale, i.e.

$${PRS}_{i}=\sum_{m=1}^{M}\hat{\beta_{m}}G_{i,m},$$

where $\hat{\beta_{m}}$ denotes the estimated log odds ratio for variant *m* and *G*_*i*,*m*_ denotes the genotype dosage of individual *i* for variant *m*. More specifically, $\hat{\beta_{m}}$ is the effect size estimate of variant *m* on a logit scale in conferring risk of CD in an individual.

In the setting where effects are non-additive, i.e. a genotype-specific effect model,

$${PRS}_{i}^{*}=\sum_{m=1}^{M}\left[\hat{\beta_{m}^{Het}}1_{\left[Het\right]}+\hat{\beta_{m}^{Hom}}1_{\left[Hom\right]}\right].$$

For now, we consider the additive scenario, and later we return to the setting where non-additive effects exist, which is relevant for quantifying the differences in contribution of *NOD2* alleles to genetic risk in two populations.

The estimated expected PRS value for an individual in population *j* is

$$E\hat{{\left[PRS\right]}_{j}}=\sum_{i=1}^{N_{j}}\frac{{PRS}_{i}}{N_{j}},$$

where *N*_*j*_ is the number of individuals sampled in population *j*. Substituting equation for *PRS*_*i*_ and rearranging terms simplifies the equation as a function of variant frequency:

$$E\hat{{\left[PRS\right]}_{j}}=\sum_{i=1}^{N_{j}}\sum_{m=1}^{M}\frac{\hat{\beta_{m}}G_{i,m}}{N_{j}},$$

$$E\hat{{\left[PRS\right]}_{j}}=\sum_{m=1}^{M}\left(\sum_{i=1}^{N_{j}}\frac{\hat{\beta_{m}}G_{i,m}}{N_{j}}\right),$$

$$E\hat{{\left[PRS\right]}_{j}}=\sum_{m=1}^{M}\left(\hat{\beta_{m}}\sum_{i=1}^{N_{j}}\frac{G_{i,m}}{N_{j}}\right),$$

where $\sum_{i=1}^{N_{j}}\frac{G_{i,m}}{N_{j}}=\hat{f_{m,j}}$ and $\hat{f_{m,j}}$ denotes the frequency of variant *m* in population *j*. Thus, the estimated expected PRS value of an individual in population *j* is $E\hat{{\left[PRS\right]}_{j}}=\sum_{m=1}^{M}\left(2\hat{\beta_{m}}\hat{f_{m,j}}\right)$.

Assume that we are interested in the expected difference in contribution of the studied variants to the PRS between two individuals, say from population 1 being AJ and population 2 being NFE. Also, assume that the effect size of variant *m* is shared across both populations. Then, using the estimated expected PRS value we define estimated expected difference in contribution of the studied variants to the PRS as the difference in estimated expected PRS value in two populations:

$$E[\hat{\mathrm{Difference}\;PRS}]=E{\hat{\left[PRS\right]}}_{AJ}-E{\hat{\left[PRS\right]}}_{NFE},$$

$$E[\hat{\mathrm{Difference}\;PRS}]=\sum_{m=1}^{M}2\hat{\beta_{m}}(\hat{f_{m,AJ}}-\hat{f_{m,NFE}}),$$

which can be used to get an estimated difference in contribution of a variant *m* to the polygenic risk score in two populations,

$${E[\hat{\mathrm{Difference}\;PRS}]}_{m}=2\hat{\beta_{m}}(\hat{f_{m,AJ}}-\hat{f_{m,NFE}}).$$

To rank variants according to their relative differences in contribution to genetic risk we included the *NOD2* and *LRRK2* alleles, used the list of estimated effect size from the published studies[36,37], and estimates from this study.

If we substitute PRS* for *PRS*,

$$E{\hat{\left[{PRS}^{*}\right]}}_{j}=\sum_{m=1}^{M}\frac{\left(\sum_{i=1}^{N_{j}}\left[\hat{\beta_{m}^{Het}}1_{\left[Het\right]}+\hat{\beta_{m}^{Hom}}1_{\left[Hom\right]}\right]\right)}{N_{j}}$$

$$=\sum_{m=1}^{M}\left[2\hat{f_{m}}(1-\hat{f_{m}})\hat{\beta_{m}^{Het}}+{\hat{f_{m}}}^{2}\hat{\beta_{m}^{Hom}}\right].$$

Then, the estimated expected difference in PRS* when non-additive effects exist is

$$E[\hat{\mathrm{Difference}\;{PRS}^{*}}]=\sum_{m=1}^{M}\left[2\hat{\beta_{m}^{Het}}(\left[\hat{f_{m}^{AJ}}-\hat{f_{m}^{NFE}}]-[{\hat{f_{m}^{AJ}}}^{2}-{\hat{f_{m}^{NFE}}}^{2}\right])+\hat{\beta_{m}^{Hom}}({\hat{f_{m}^{AJ}}}^{2}-{\hat{f_{m}^{NFE}}}^{2})\right].$$

### Estimating fold difference in prevalence for a population with shift in expected genetic risk

Assuming log-additive effects in the logit risk model the disease probability for an individual is given as *p* = (1 + exp(−*η*))^−1^, where η tends towards a normal distribution with parameters $\mu =\log(p_{0}/(1-p_{0}))+\sum_{m=1}^{M}2f_{m}\beta_{m}$ and $\sigma^{2}=2\sum_{m=1}^{M}f_{m}(1-f_{m})\beta_{m}^{2}$ [35]. Here *p*_*0*_ refers to a baseline disease probability.

We can see that μ may be expressed in terms of the expected polygenic risk score, i.e. $\mu =\log(p_{0}/(1-p_{0}))+E[PRS]$. In the setting where E[PRS]=0, then

$$E[p]={\left(1+\exp(-\log(p_{0}/(1-p_{0})))\right)}^{-1}=p_{0}.$$

To evaluate the impact of a shift in the expected value of polygenic risk score to the expected value of *μ* we can express the shift as E[Differenceμ]=E[DifferencePRS]. We can compute new values of *p* for new values of *μ* to obtain a fold-increase in prevalence for a population that has undergone such a shift.

We see that this requires a value to be chosen for *p*_*0*_ and that log(*p*_0_/(1 − *p*_0_)) can be represented as a baseline risk score value β_*0*._To get an estimate of the absolute prevalence of CD in the AJ population, we must choose a baseline β_*0*,_ where *p*_*0*_ represents the expected prevalence with zero non-baseline alleles in the population[35], to which we add a contribution from multiple non-baseline alleles to calculate: 1) an individual’s probability of disease, or 2) the expected prevalence of the disease in the population.

Once we have chosen a value for β_*0*_, we can calculate the ratio of expected prevalence as follows. First, use the means (μ_AJ_ and μ_NAJ_) and variances ($\sigma_{AJ}^{2}$ and $\sigma_{NAJ}^{2}$) of risk scores as calculated above to calculate the probability density function of the disease prevalence. In the case of the AJ population, we have

$$f(p)=\frac{dn}{dg}\frac{1}{\sigma_{AJ}}\phi \left(\frac{\eta -\mu_{AJ}}{\sigma_{AJ}}\right)=\frac{1}{\sigma_{AJ}p(1-p)}\phi \left(\frac{1}{\sigma_{AJ}}\log(\frac{p}{1-p})-\frac{\mu_{AJ}}{\sigma_{AJ}}\right)$$

where η is the risk score associated with prevalence *p*, *g* is the link function, so *p* = *g*(*η*) = (1 + *e*^−*η*^)^−1^, and ϕ is the standard normal density function.

Next, we integrate to get $\int_{0}^{1}p∙f(p)dp=E[p_{AJ}]$. Finally, we can calculate $E[p_{NAJ}]$ in a similar way, and divide the expected prevalence in the AJ population by that in the non-AJ population to get the prevalence ratio, $E[p_{AJ}]/E[p_{NAJ}]$.

The value of β_*0*_ = -20.5 was chosen in order to obtain a prevalence in the non-AJ population of ~0.5%. At this value of β_*0*_, the ratio of prevalence in the AJ population to that in the non-AJ population was estimated to be 1.5 ($E[p_{AJ}]$ = 0.82%, $E[p_{NAJ}]$ = 0.55%).

For different choices of β_*0*_, however, this ratio may vary, as the relationship between probability of disease and risk score is non-linear. S10 Fig shows how the values of the disease prevalence and their ratio vary as β_*0*_ is changed. We see that the ratio values range from 1.46 to 1.52 for different values of β_*0*_ with a range of baseline prevalence of .001 to .01—the range of prevalence estimates for Crohn’s disease[41,43,57].

To further understand the effect that choosing a logit-based model had on the results, a comparison of the standard logit and probit models was done using the values inferred from the logit model. No full scale probit modelling was done in this analysis, so the values found with the probit model represent only a close approximation of the expected results.

In the logit model for population analysis, we may assume that individual risk scores are chosen from a normal distribution $N(\mu_{\mathrm{logit}},\;\sigma_{\mathrm{logit}}^{2})$ where μ_logit_ and *σ*_logit_ represent the mean and standard deviation of the risk scores as defined above. From here, we may calculate the probability density function of probit model risk scores μ_probit_ based on that of logit model risk scores μ_logit_ as

$$f(\eta_{\mathrm{probit}}|\mu_{\mathrm{logit}},\;\sigma_{\mathrm{logit}})=f(\eta_{\mathrm{logit}}|\mu_{\mathrm{logit}},\;\sigma_{\mathrm{logit}}){d\eta }_{\mathrm{logit}}/{d\eta }_{\mathrm{probit}}$$

and use this to calculate μ_probit_ and *σ*_probit_, the estimated mean and standard deviation of the risk scores in the probit model. Using these values, we obtain a probability distribution for the frequency of disease in the populations using the probit model.

While the logit model yielded a prevalence ratio of 1.506, the probit estimation yielded a prevalence ratio of 1.5136, with similar expected prevalence values ($E[p_{AJ}]$ = 0.823%, $E[p_{NAJ}]$ = 0.544%). These values demonstrate that individual logit and probit analyses would likely give similar results for values of interest. The complete probability densities under the logit and probit models can be seen in S8 Fig.

Further, it is interesting to compare the relationship between values of risk scores in the two models. For values of risk scores between -1 and 1 in the logit model, the relationship to those in the probit model is highly linear, with a formula of *η*_probit_ = 0.6223 ∙ *η*_logit_, with r^2^ = 1.0000. This formula may be used to impute single values in one model or the other assuming that the estimated total risk score is otherwise close to zero, and the imputed value is low. It is worth noting, however, that this does not work for all values of η_logit_, as the relationship between risk score in the logit and probit models deviates from this simple linear model when the risk score values are large.

#### Difference in prevalence between AJ and NFE attributed to implicated variants

The difference in prevalence due to multiple alleles can be computed as

$$Prevalence\;difference=\frac{\left(\left(p_{2}-p_{1})-(i_{2}-i_{1}\right)\right)}{\left(p_{2}-p_{1}\right)},$$

where *p*_*j*_ denotes the disease prevalence in population *j* and *i*_*j*_ denotes the disease prevalence without the risk factors in population *j*, which according to Moonesinghe et al.[58] is

$$i_{j}=\frac{p_{j}}{\prod_{m=1}^{M}{\left(1+f_{m,j}({GRR}_{m}-1)\right)}^{2}}$$

where *GRR*_*m*_ denotes the genotype relative risk for variant *m*.

We model the CD prevalence accounted for by CD associated enriched protein-altering alleles separately in both AJ and non-AJ European and determine the amount that CD prevalence would be reduced if this variant were absent from each population.

To estimate the difference in prevalence between two populations attributed to genetic risk factors when non-additive effects exist,

$$i_{j}=\frac{p_{j}}{\prod_{m=1}^{M}\left(1+2f_{m,j}({GRR}_{m}^{Het}-1)+f_{m,j}^{2}({GRR}_{m}^{Hom}-1)\right)}.$$

### Enirchment testing sensitivity

When modeling enrichment, we chose a standard significance cutoff of p < 0.05/N for classifying variants as enriched. We noted that the number of variants classified as enriched does not change significantly when the p-value threshold changes. See S11 Fig for more information.

## Supporting information

S1 Fig Analysis workflow diagram. (PNG)

S2 Fig Admixture plots. (PNG)

S3 Fig Cross-validation errors for number of clusters in ADMIXTURE. (PNG)

S4 Fig MAF thresholds chosen for enrichment testing. (PNG)

S5 Fig Principal components plot for 5,685 AJ individuals. (PNG)

S6 Fig Variable selection using Bayesian model averaging (BMA). (PNG)

S7 Fig AJ individuals have higher CD polygenic risk score than NJ controls. (PNG)

S8 Fig Dependence of population prevalence on *β_0_*. (PNG)

S9 Fig Probit and logit model analysis. (PNG)

S10 Fig The relationship between expected differences in genetic risk score and expected fold differences in disease prevalence. (PNG)

S11 Fig Sensitivity test for p-value significance cutoff. (PNG)

S1 Data File ClinVar pathogenic alleles enriched in AJ. (XLSX)

S2 Data File AJ enrichment data for all analyzed alleles. (TXT)

S1 Table Origins of moderate ancestry fraction Ashkenazi samples. (PNG)

S2 Table Origins of high ancestry fraction Ashkenazi samples. (PNG)

S3 Table Conditional haplotype-based testing in *NOD2*. (PNG)

S4 Table Assessing association of a one-hit and two-hit model of *NOD2* in the AJ exome sequencing data. (PNG)

S5 Table Assessing association of a one-hit and two-hit model of *NOD2* in the non-AJ immunoChip data. (JPG)
