# Correction: Insights into the genetic epidemiology of Crohn's and rare diseases in the Ashkenazi Jewish population

**DOI:** 10.1371/journal.pgen.1008190

**Published:** 2019-05-30

**Authors:** Manuel A. Rivas, Brandon E. Avila, Jukka Koskela, Hailiang Huang, Christine Stevens, Matti Pirinen, Talin Haritunians, Benjamin M. Neale, Mitja Kurki, Andrea Ganna, Daniel Graham, Benjamin Glaser, Inga Peter, Gil Atzmon, Nir Barzilai, Adam P. Levine, Elena Schiff, Nikolas Pontikos, Ben Weisburd, Monkol Lek, Konrad J. Karczewski, Jonathan Bloom, Eric V. Minikel, Britt-Sabina Petersen, Laurent Beaugerie, Philippe Seksik, Jacques Cosnes, Stefan Schreiber, Bernd Bokemeyer, Johannes Bethge, International IBD Genetics Consortium, NIDDK IBD Genetics Consortium, T2D-GENES Consortium, Graham Heap, Tariq Ahmad, Vincent Plagnol, Anthony W. Segal, Stephan Targan, Dan Turner, Paivi Saavalainen, Martti Farkkila, Kimmo Kontula, Aarno Palotie, Steven R. Brant, Richard H. Duerr, Mark S. Silverberg, John D. Rioux, Rinse K. Weersma, Andre Franke, Luke Jostins, Carl A. Anderson, Jeffrey C. Barrett, Daniel G. MacArthur, Chaim Jalas, Harry Sokol, Ramnik J. Xavier, Ann Pulver, Judy H. Cho, Dermot P. B. McGovern, Mark J. Daly

The data in the S2 Data File does not display correctly. Please view the correct S2 Data File below.

## Supporting information

S2 Data File AJ enrichment data for all analyzed alleles. (XLSX)
