# Supplementary figures and images for: Insights into the genetic epidemiology of Crohn's and rare diseases in the Ashkenazi Jewish population
Source: PLoS Genet. 2018 May 24;14(5):e1007329. doi: 10.1371/journal.pgen.1007329 (PMC5967709; doi:10.1371/journal.pgen.1007329)

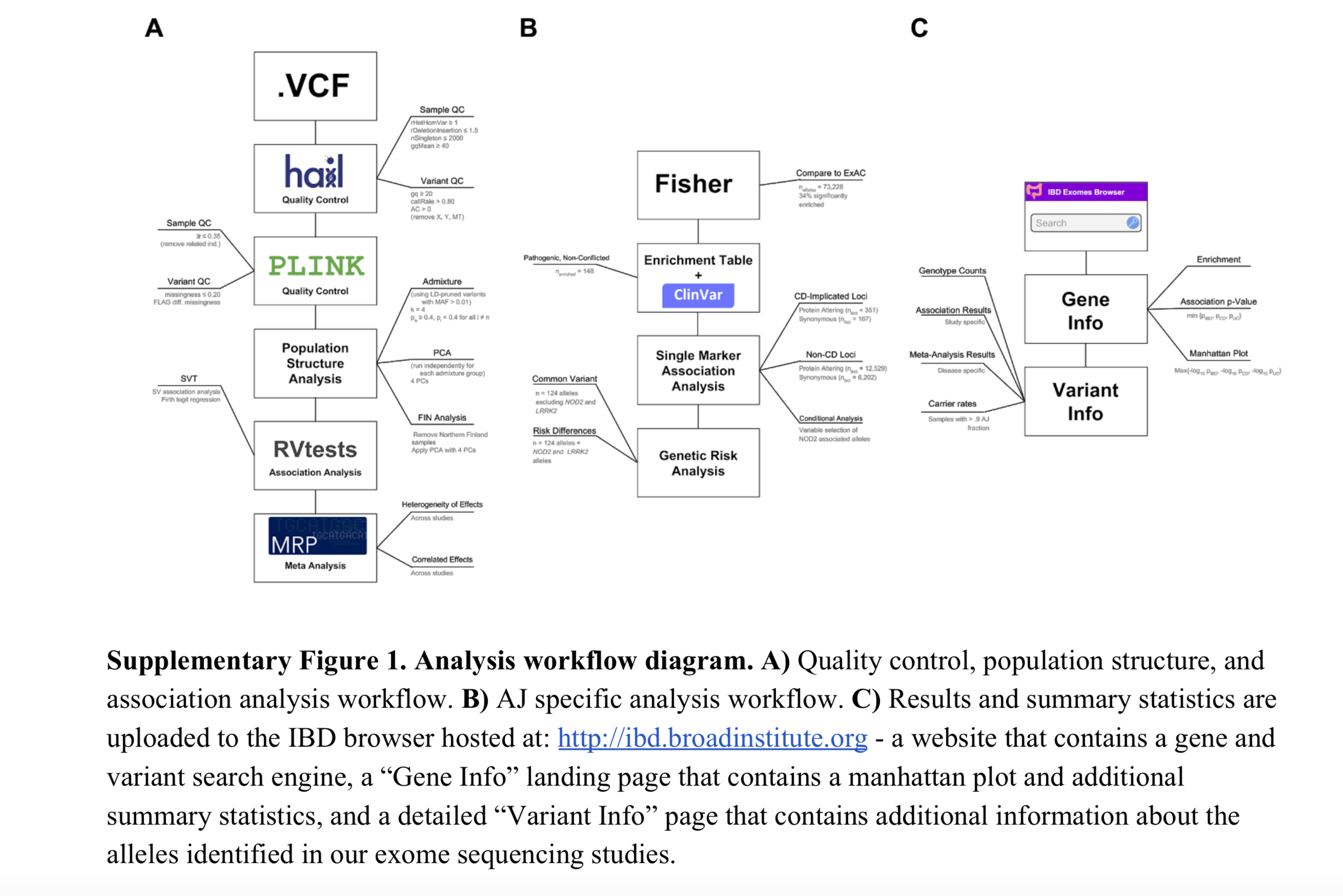

Supplement: S1 Fig — (PNG) [file pgen.1007329.s001.png]

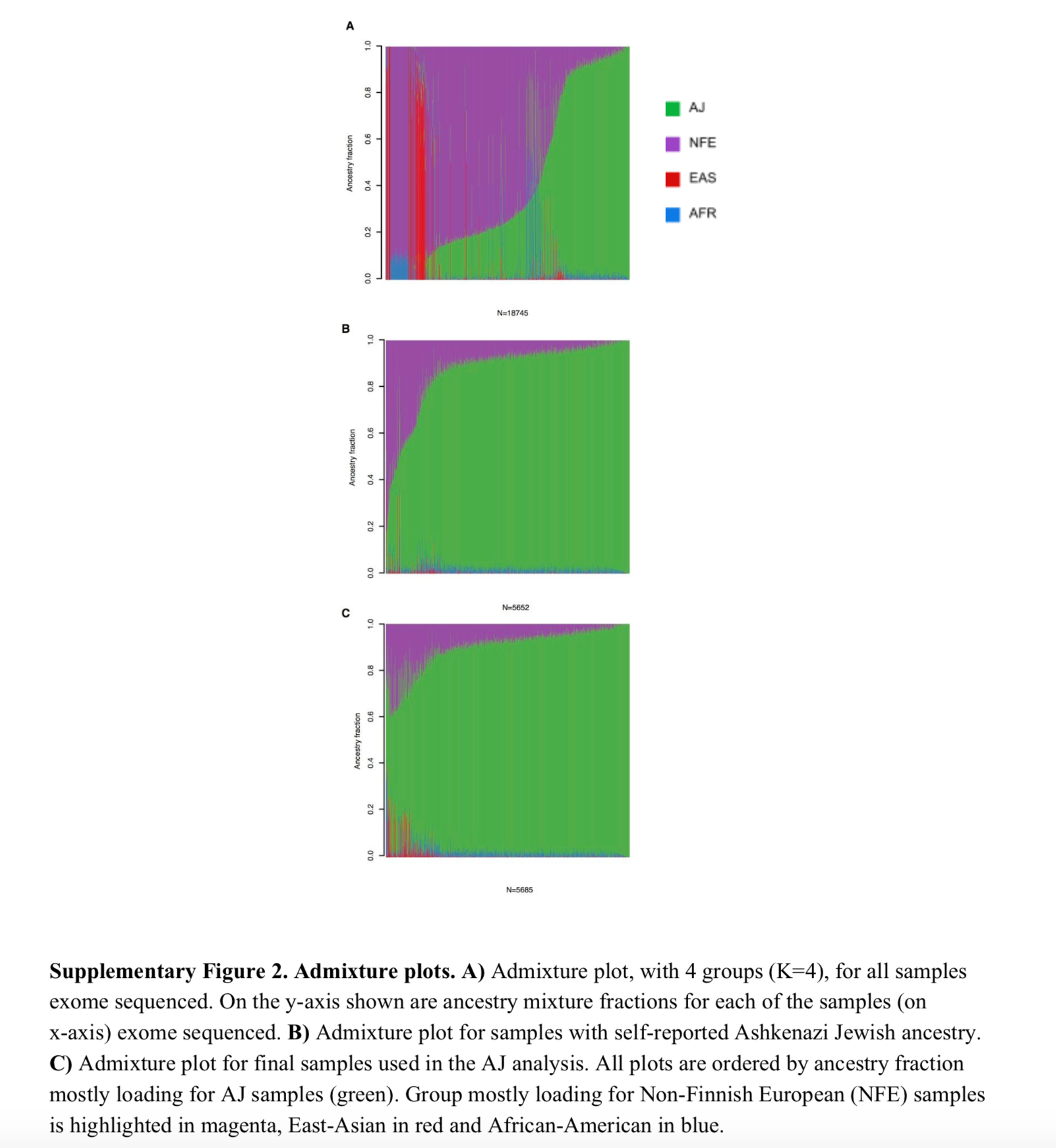

Supplement: S2 Fig — (PNG) [file pgen.1007329.s002.png]

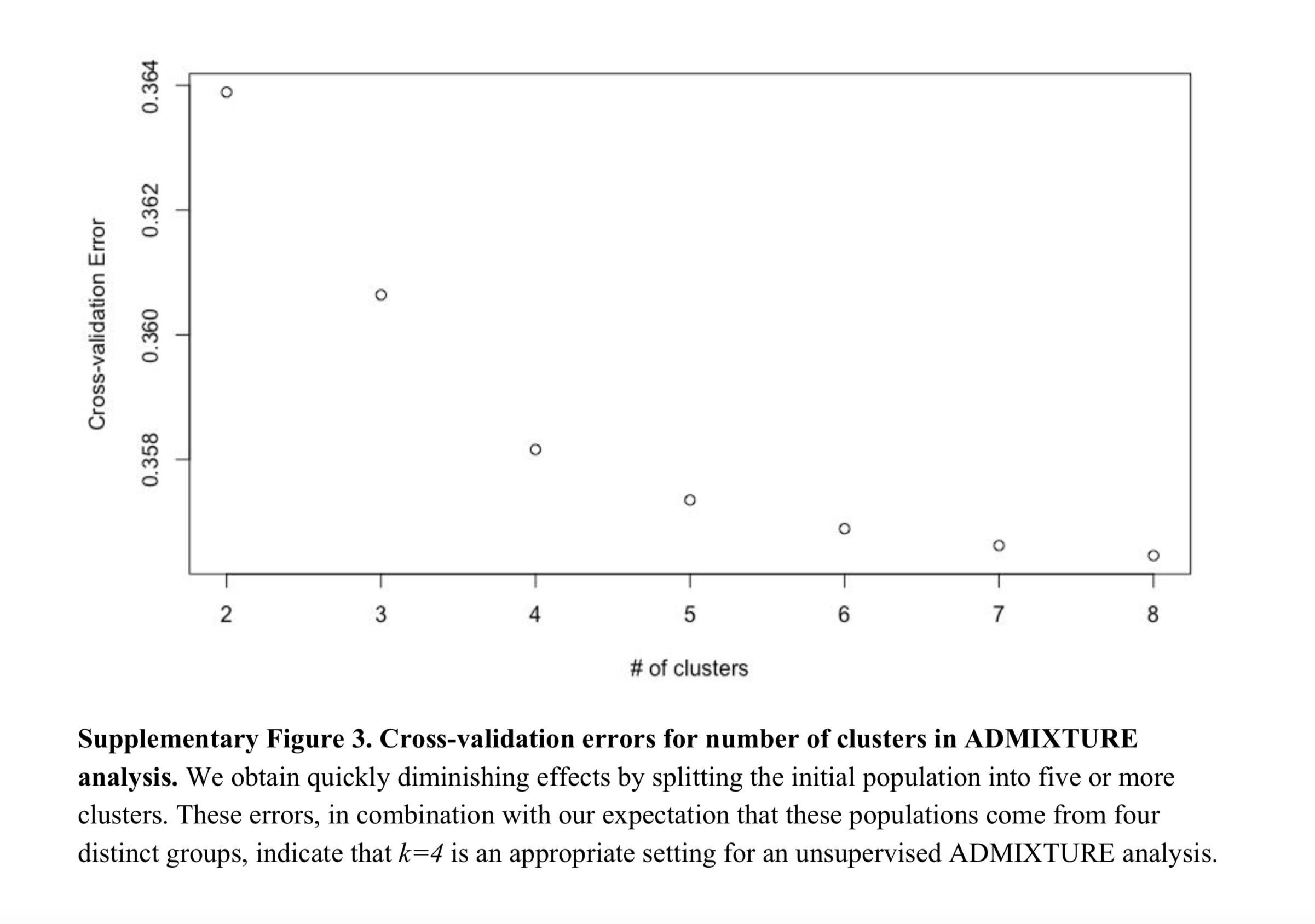

Supplement: S3 Fig — (PNG) [file pgen.1007329.s003.png]

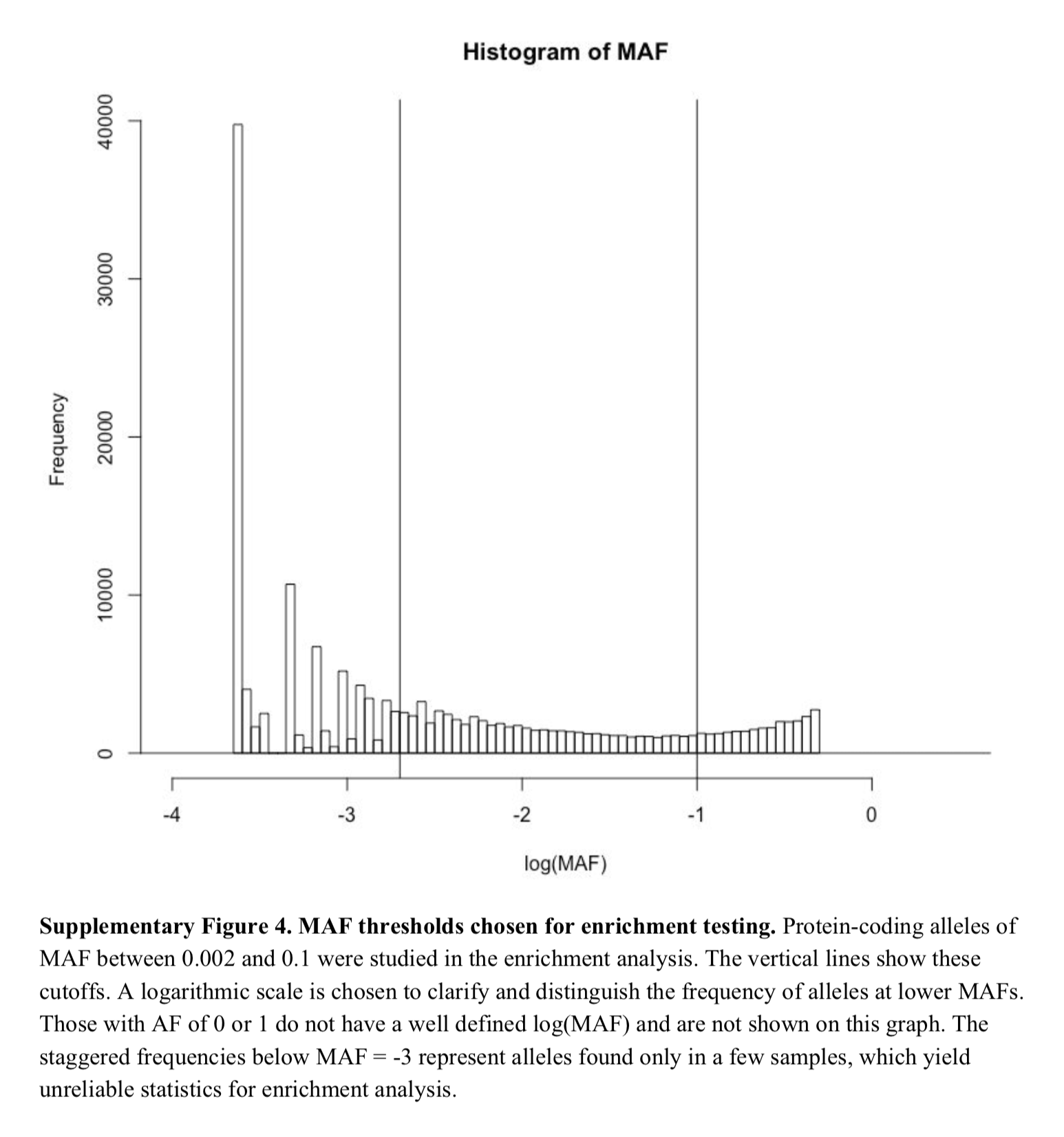

Supplement: S4 Fig — (PNG) [file pgen.1007329.s004.png]

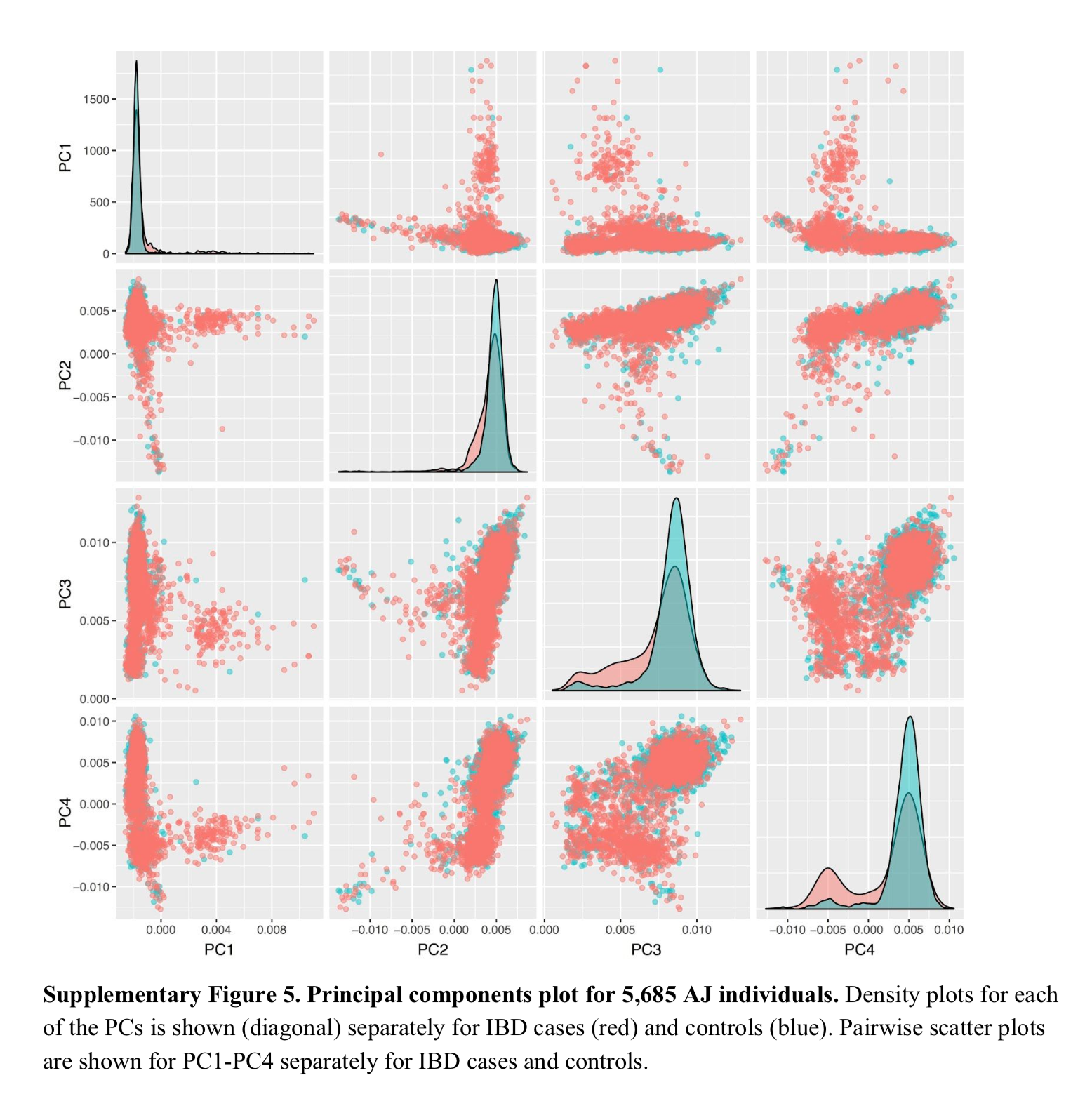

Supplement: S5 Fig — (PNG) [file pgen.1007329.s005.png]

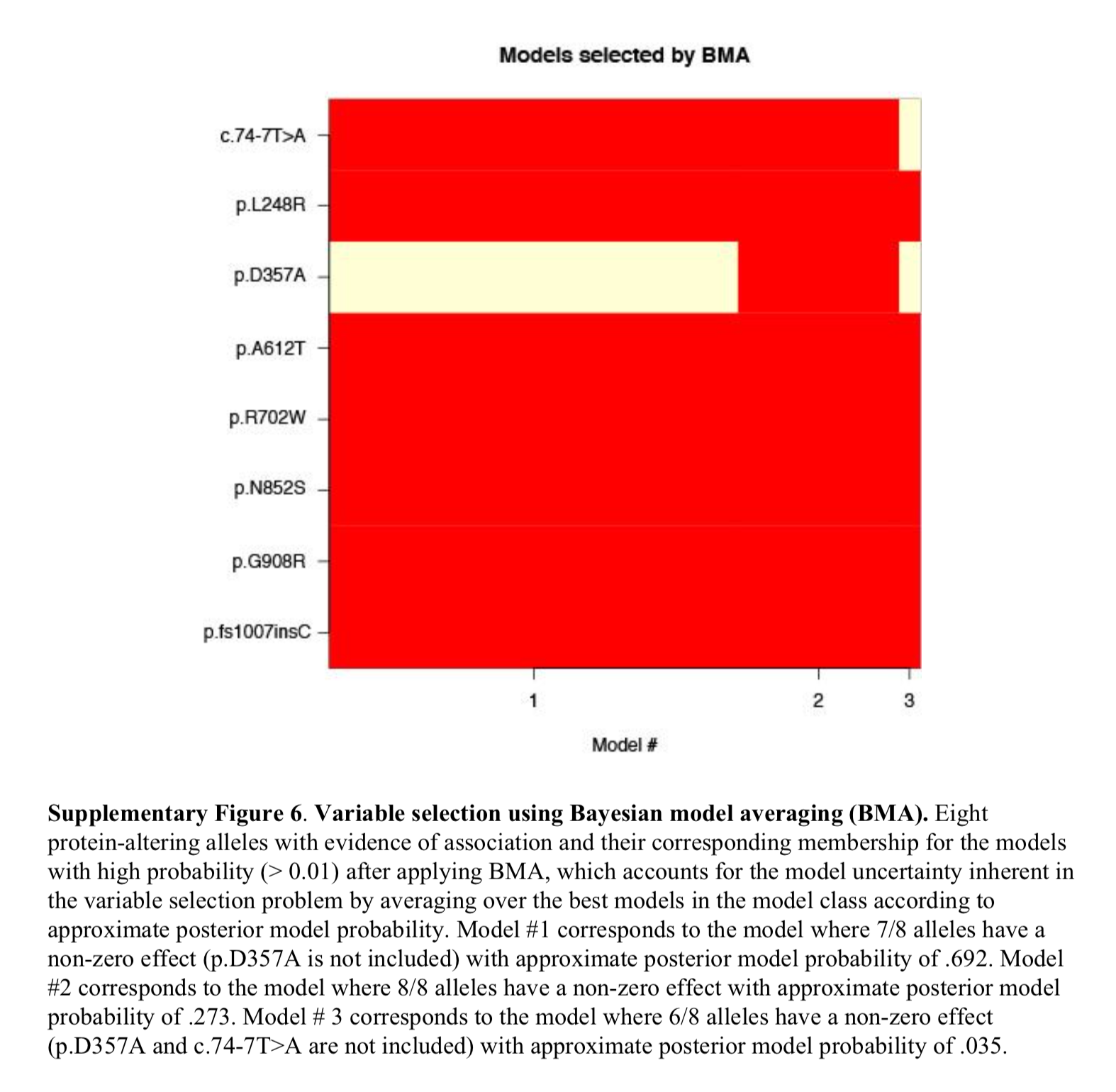

Supplement: S6 Fig — (PNG) [file pgen.1007329.s006.png]

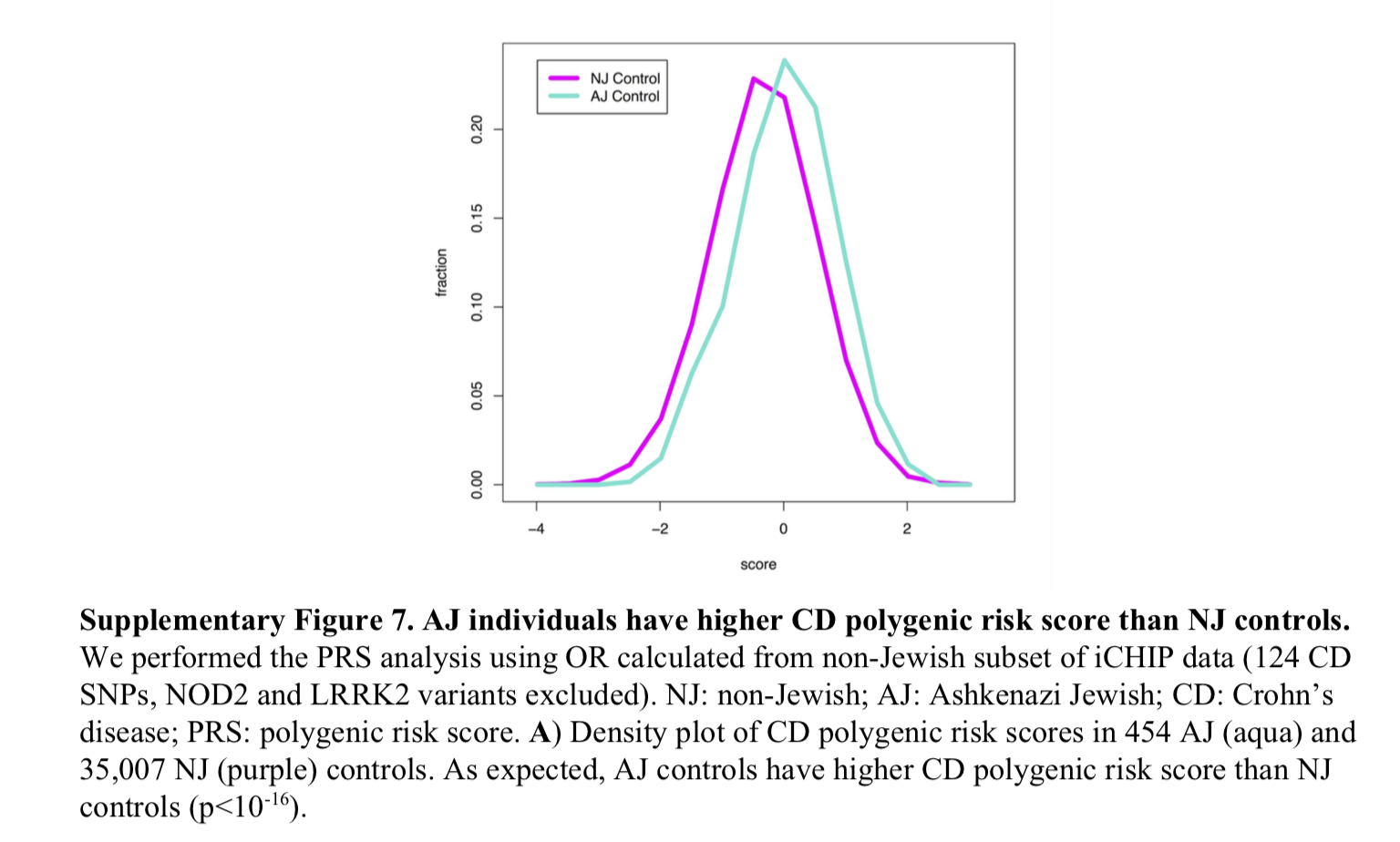

Supplement: S7 Fig — (PNG) [file pgen.1007329.s007.png]

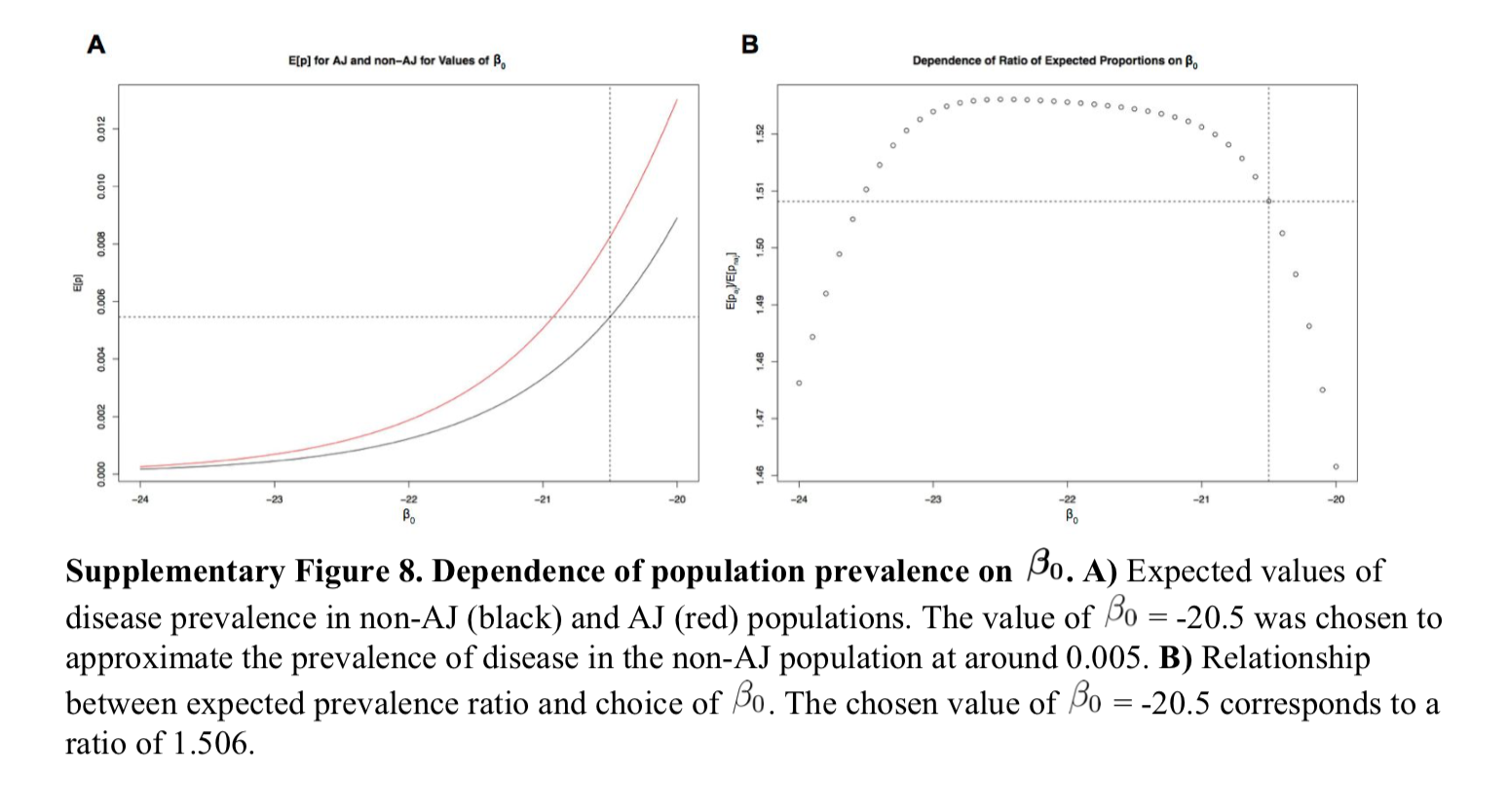

Supplement: S8 Fig — (PNG) [file pgen.1007329.s008.png]

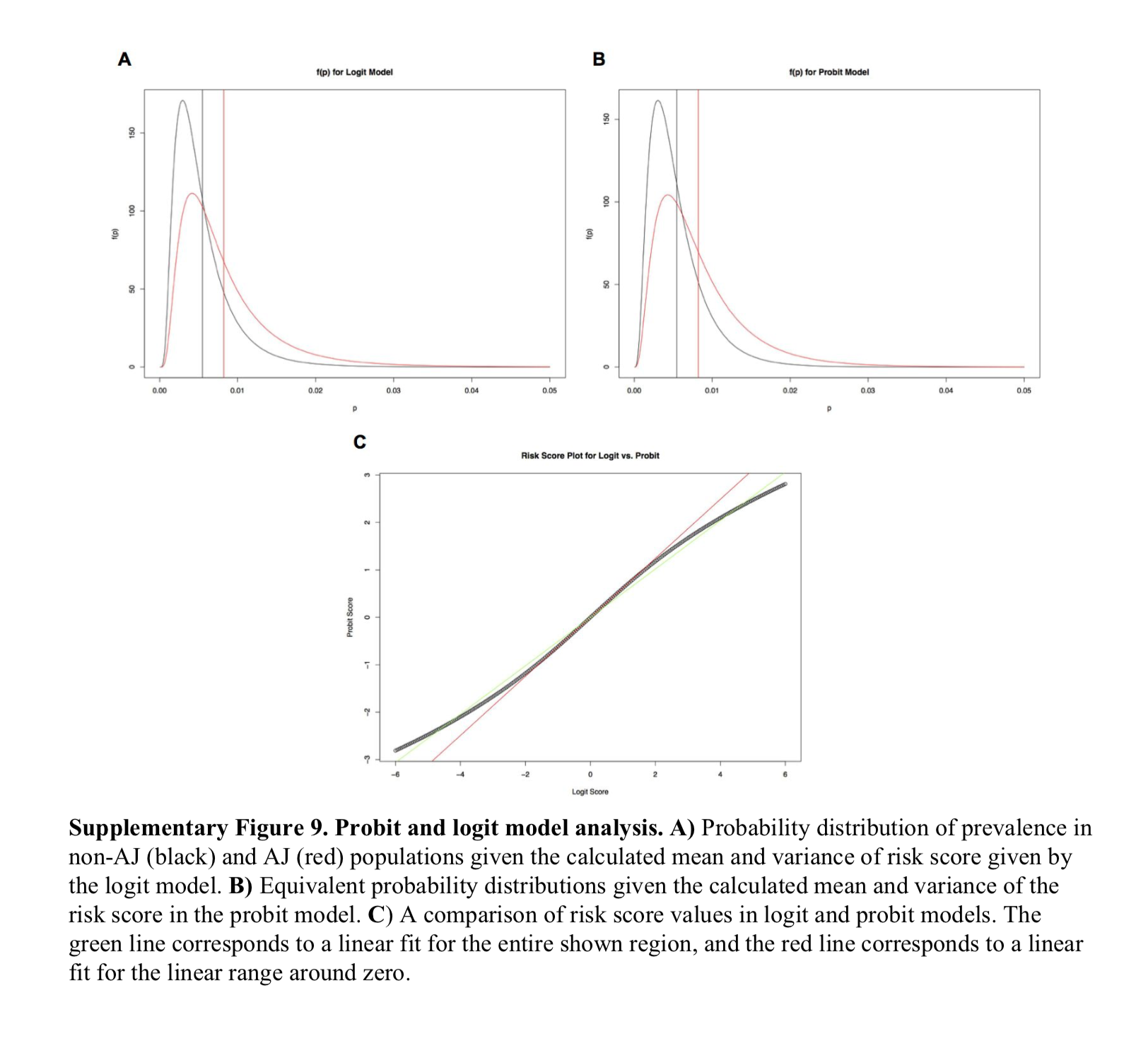

Supplement: S9 Fig — (PNG) [file pgen.1007329.s009.png]

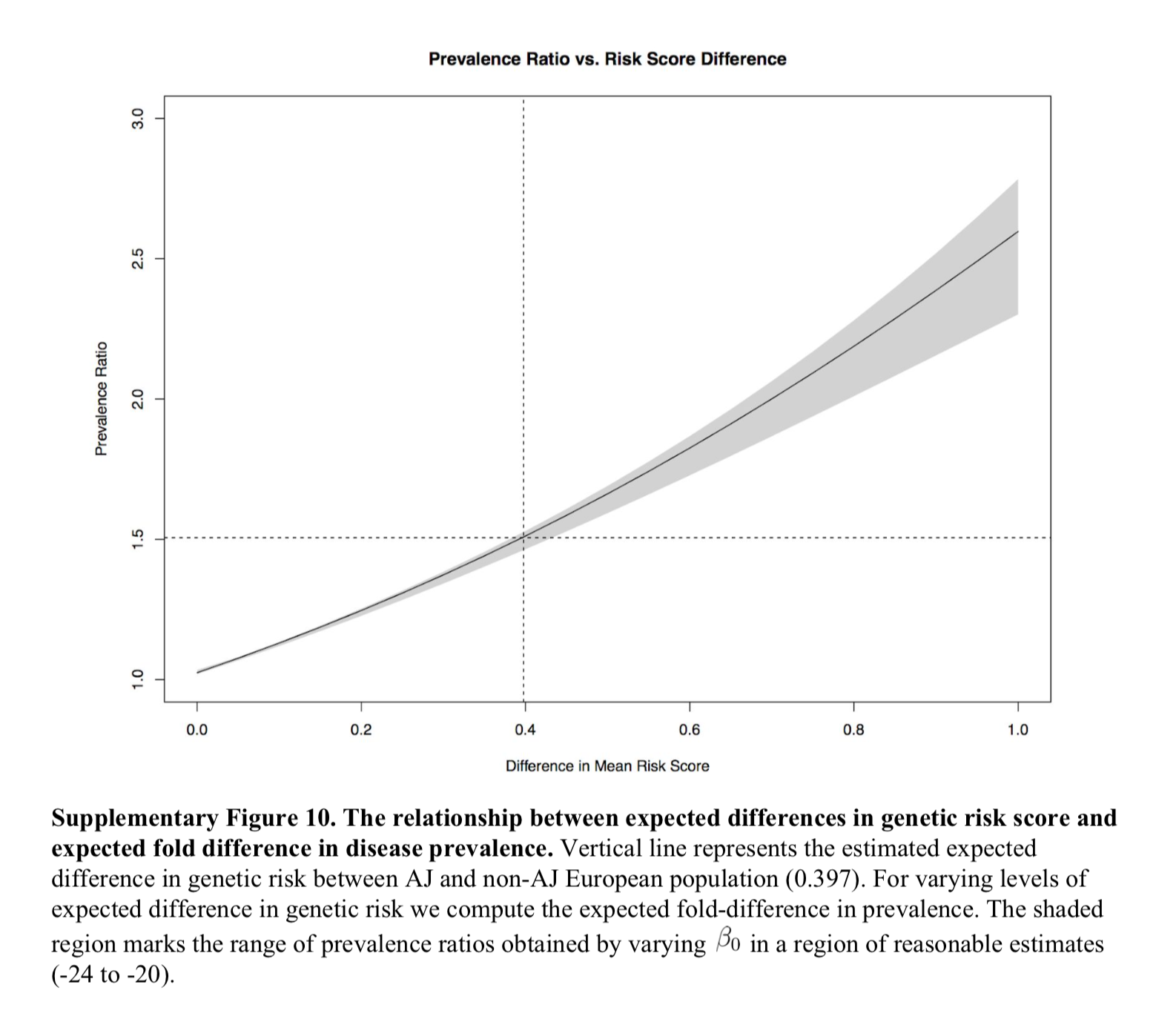

Supplement: S10 Fig — (PNG) [file pgen.1007329.s010.png]

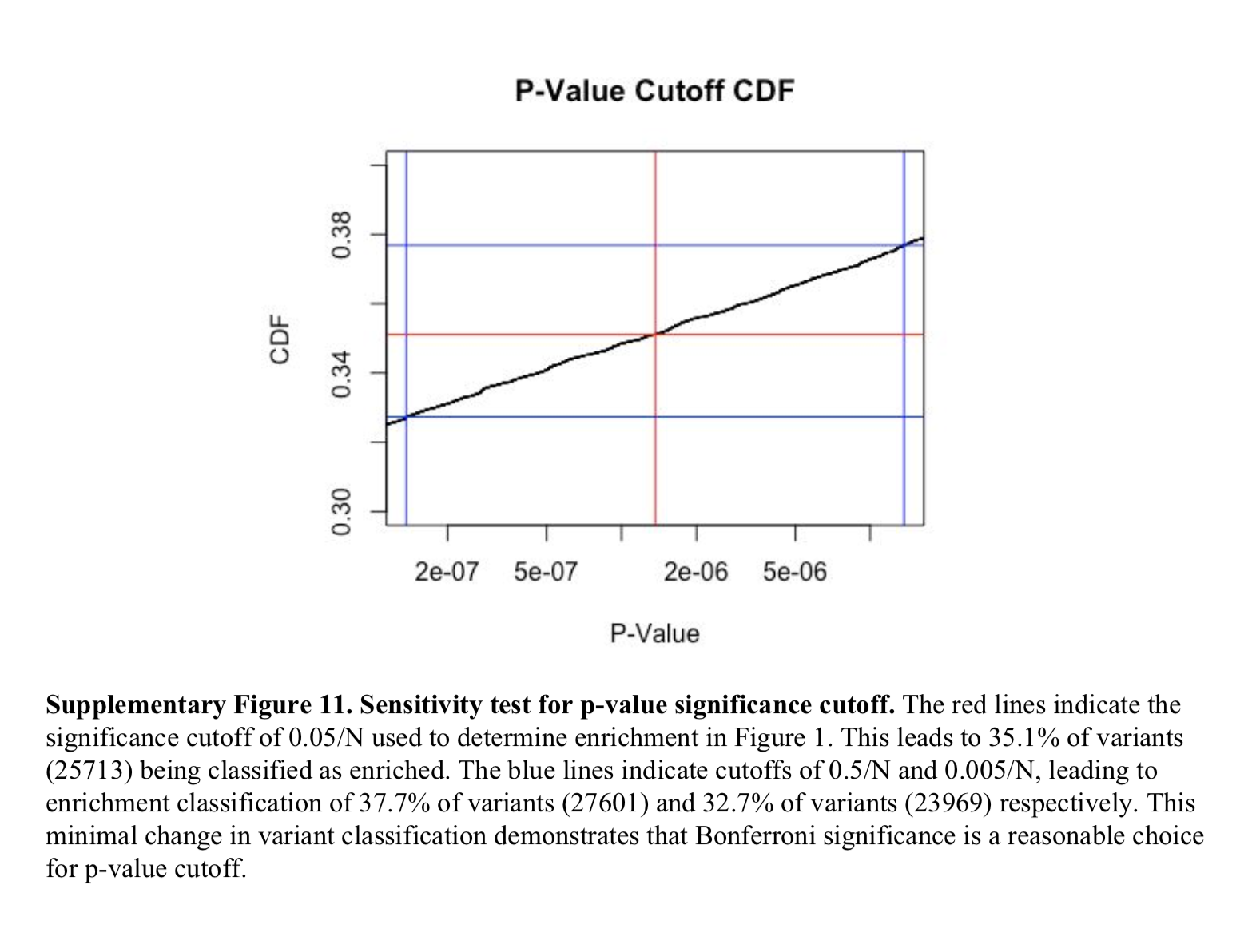

Supplement: S11 Fig — (PNG) [file pgen.1007329.s011.png]

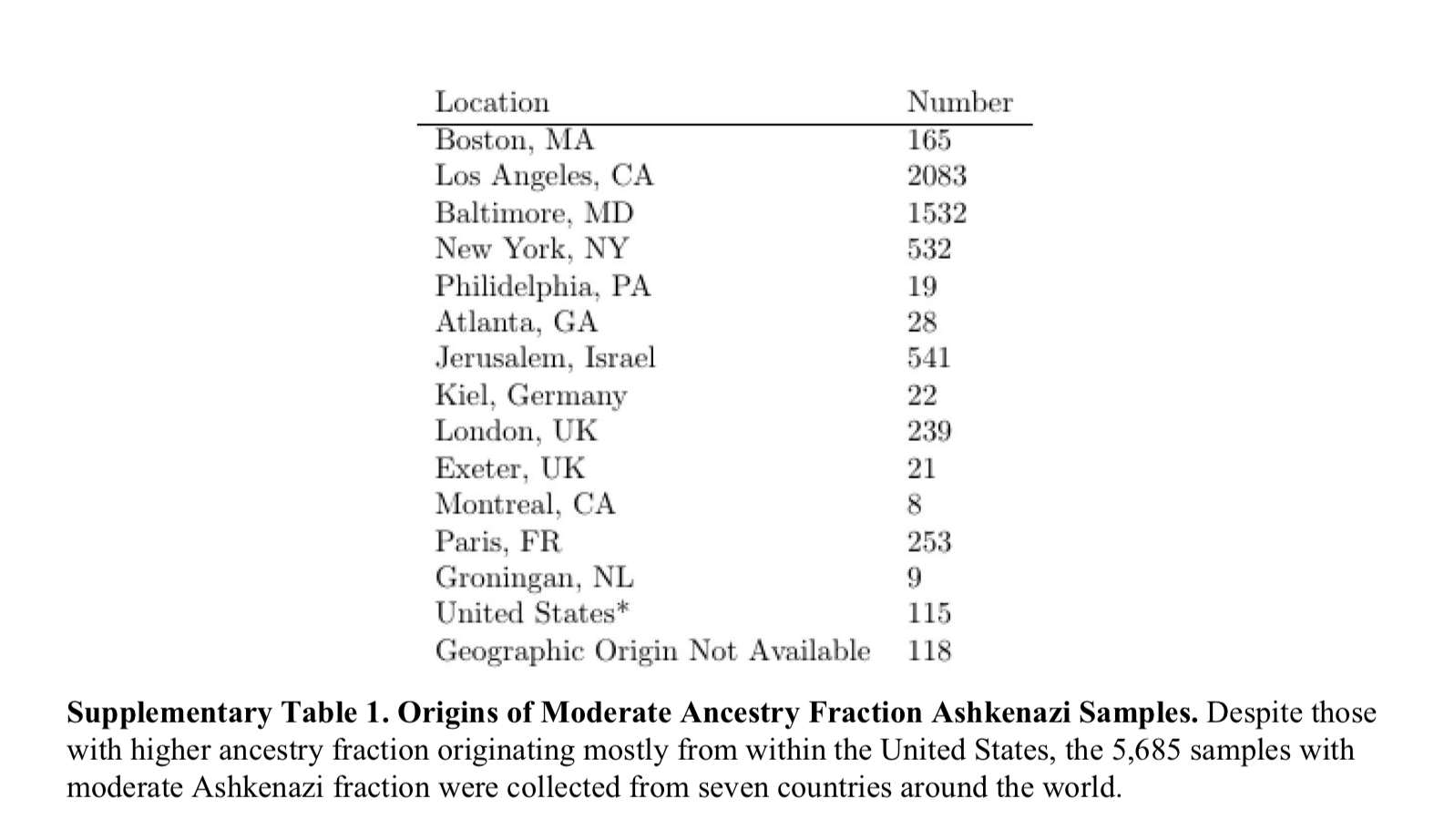

Supplement: S1 Table — (PNG) [file pgen.1007329.s014.png]

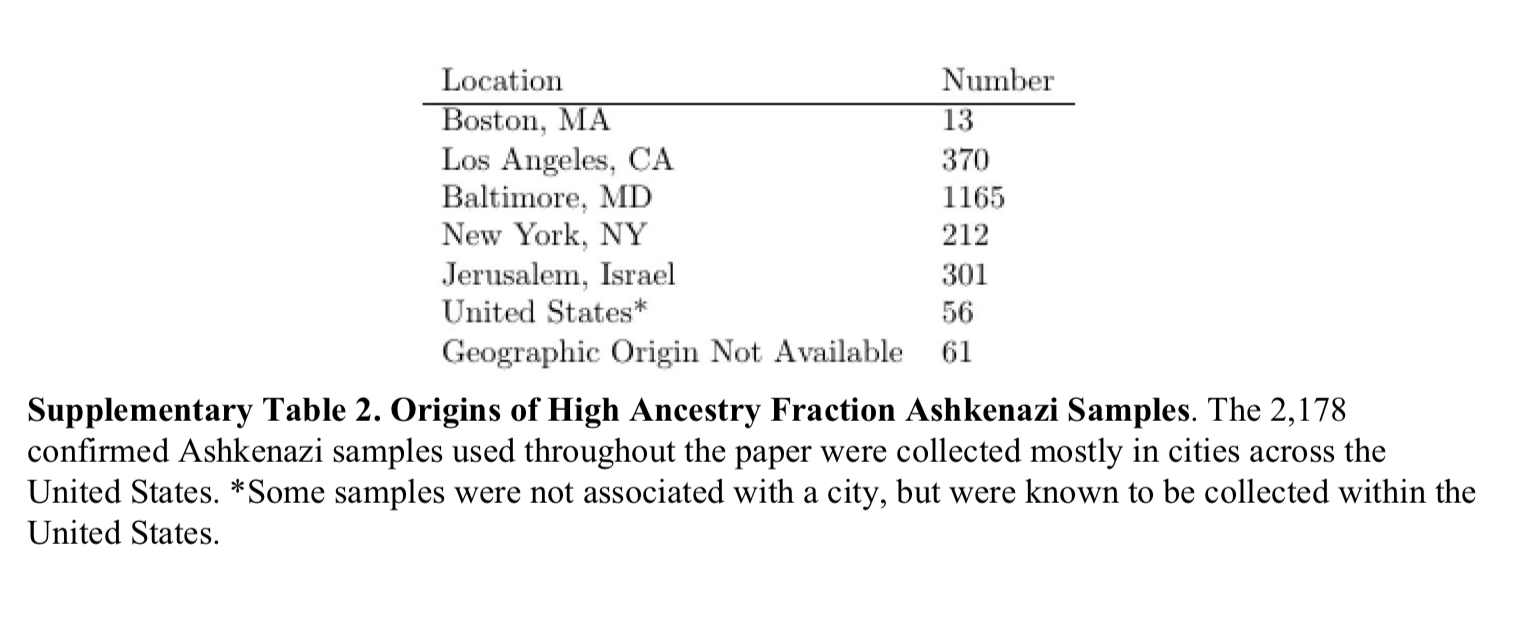

Supplement: S2 Table — (PNG) [file pgen.1007329.s015.png]

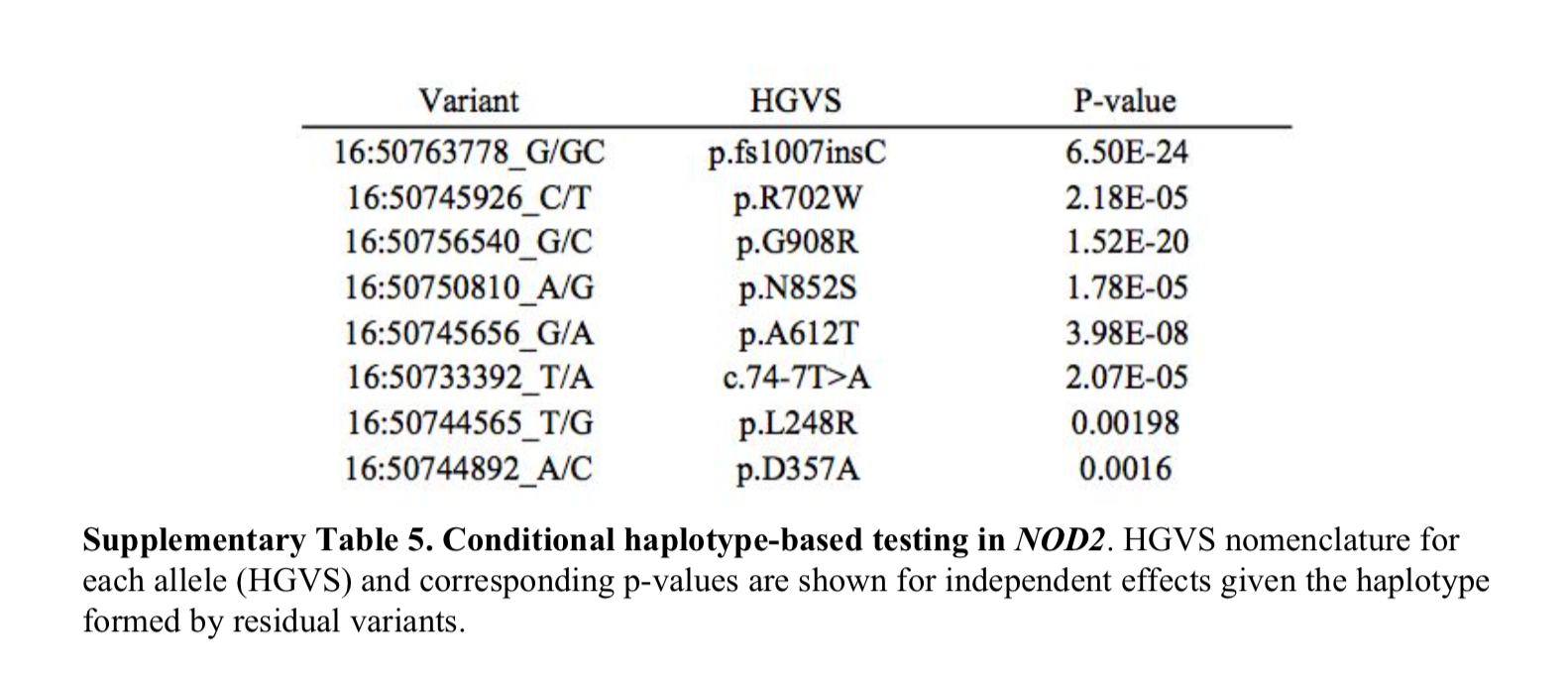

Supplement: S3 Table — (PNG) [file pgen.1007329.s016.png]

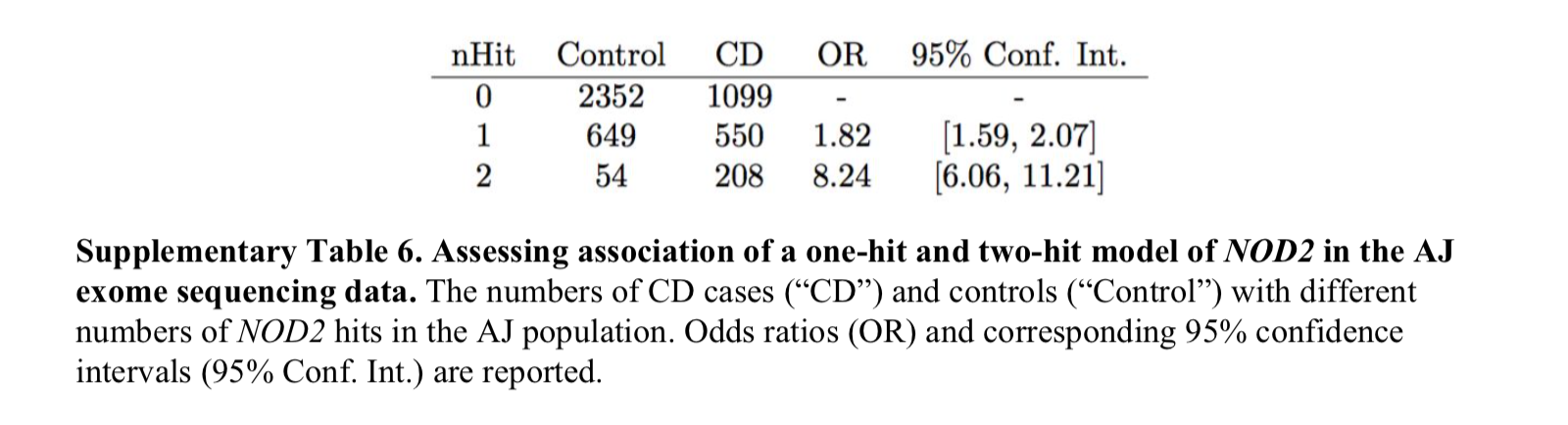

Supplement: S4 Table — (PNG) [file pgen.1007329.s017.png]

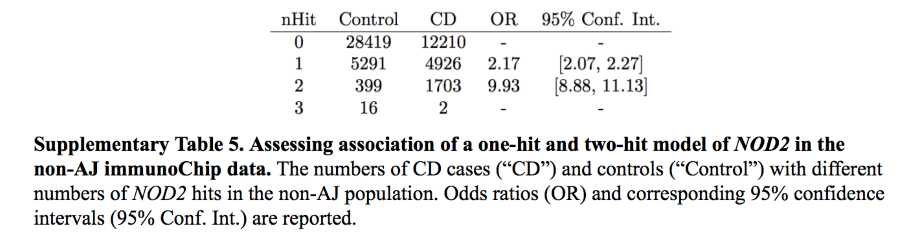

Supplement: S5 Table — (JPG) [file pgen.1007329.s018.jpg]
